# Transcriptomic analysis of *Citrus clementina* mandarin fruits maturation reveals a MADS-box transcription factor that might be involved in the regulation of earliness

**DOI:** 10.1186/s12870-019-1651-z

**Published:** 2019-01-31

**Authors:** Javier Terol, M. José Nueda, Daniel Ventimilla, Francisco Tadeo, Manuel Talon

**Affiliations:** 1 Centro de Genómica, Instituto Valenciano de Investigaciones Agrarias (IVIA), 46113 Moncada, Valencia Spain; 2 Facultad de Ciencias, Universidad de Alicante, Alicante, Spain

**Keywords:** Citrus, RNA-Seq, Fruit, Ripening, Earliness, Lateness, Transcription factor, MADS box

## Abstract

**Background:**

Harvest time is a relevant economic trait in citrus, and selection of cultivars with different fruit maturity periods has a remarkable impact in the market share. Generation of early- and late-maturing cultivars is an important target for citrus breeders, therefore, generation of knowledge regarding the genetic mechanisms controlling the ripening process and causing the early and late phenotypes is crucial. In this work we analyze the evolution of the transcriptome during fruit ripening in 3 sport mutations derived from the Fina clementine (Citrus clementina) mandarin: Clemenules (CLE), Arrufatina (ARR) and Hernandina (HER) that differ in their harvesting periods. CLE is considered a mid-season cultivar while ARR and HER are early- and late-ripening mutants, respectively.

**Results:**

We used RNA-Seq technology to carry out a time course analysis of the transcriptome of the 3 mutations along the ripening period. The results indicated that in these mutants, earliness and lateness during fruit ripening correlated with the advancement or delay in the expression of a set of genes that may be implicated in the maturation process. A detailed analysis of the transcription factors known to be involved in the regulation of fruit ripening identified a member of the MADS box family whose expression was lower in ARR, the early-ripening mutant, and higher in HER, the late-ripening mutant. The pattern of expression of this gene during the maturation period was basically contrary to those of the ethylene biosynthetic genes, SAM and ACC synthases and ACC oxidase. The gene was present in hemizygous dose in the early-ripening mutant.

**Conclusions:**

Our analysis provides new clues about the genetic control of fruit ripening in citrus and allowed the identification of a transcription factor that could be involved in the early phenotype.

**Electronic supplementary material:**

The online version of this article (10.1186/s12870-019-1651-z) contains supplementary material, which is available to authorized users.

## Background

Fruit maturity date is an important economic trait and selection of cultivars with different harvest time is desirable as it allows to extend their selling period, which can have a beneficial impact in the market share. Currently for most citrus, harvest time is mainly from November to December, which results in huge market pressure and usually in the drop of pricing. Thus, breeding of early- and late-maturing citrus cultivars is critical to extend marketing season, meet the needs of consumers and ensure an optimal adaptation to climatic and geographic conditions.

Development of citrus fruits can be divided into three stages: in the initial phases I and II fruits develop and grow, while in the final phase III, growth is mostly halted and fruits undergo a non-climacteric ripening process [1]. Color break, the key metabolic event of external ripening, takes place during phase III [2, 3]; while internal quality traits are developed along phases II and III [4]. Citrus fruits accumulate a large amount of organic acids in the vacuoles of the juice sac cells during the first half of phase II, that are gradually catabolized during the second half of phases II and III [5]. The decline in titrable acidity is due to the utilization of citric acid, the most abundant organic acid in citrus juice [6]. The ripening of citrus fruit is accompanied by carbohydrate build-up, and the major increase occurs during the acid decline stage, and towards fruit maturation, so in the end sugars accounts for 70–80% of the total soluble solids (TSS, BRIX) [7].

Color break is a process of particular economic importance, since the external color of citrus fruits is a critical quality parameter for the fresh market. Peel degreening is the result of the degradation of chlorophylls and the simultaneous accumulation of carotenoids, which confer the ripe fruits their characteristic orange color [8]. Color change is under the control of a network of regulatory metabolic signals, including ripening inducers such as ethylene and sucrose and ripening retardants, including gibberellins and nitrogen [2].

Although citrus fruits are classically considered as non-climacteric, due to the virtual absence of an increase in ethylene production and respiration rate during ripening [9], application of exogenous ethylene accelerates color changes in the peel of fruits of most Citrus species and cultivars [10]. Postharvest degreening with exogenous ethylene is commercially used worldwide to uniform and promote external coloration, especially in early-season cultivars in which the internal quality acceptable for marketability is reached when the peel is still green [8].

Transcriptional control of fruit ripening has been thoroughly studied, particularly in species like tomato (*Solanum esculentum*) that has become a model species [11]. In tomato, ripening is regulated by a number of transcription factors in conjunction with the plant hormone ethylene. Tomato fruit patterning, determinacy, and early development is regulated by SQUAMOSA promoter binding protein-like (SPL/SBP) transcription factors [12]. Later, ripening is controlled by the transcription factors NON-RIPENING (NOR) [13], COLORLESS NON-RIPENING (CNR) [14], and RIPENING INHIBITOR (MADS-RIN) [15] in concert with ethylene signaling, possibly in response to a developmental switch. Additional components include TOMATO AGAMOUS-LIKE1 (TAGL1) [16], APETALA2a (AP2a) [17], and FRUITFULL (FUL1 and FUL2) [18]. The links between this highly connected regulatory network and downstream effectors modulating color, texture, and flavor are still relatively poorly understood [19].

Of special interest regarding our work, is a tomato MADS-box gene, SlMADS1, that is highly expressed in sepals and fruits; with expression level increased with the development of sepals, but decreasing sharply as fruit ripening advances. RNA interference experiments showed shorter ripening time of fruit in SlMADS1-silenced tomatoes, with enhanced accumulation of carotenoids and upregulation of ethylene biosynthetic genes and the ethylene-responsive genes E4 and E8. These results suggest that SlMADS1 plays an important role in fruit ripening as a repressive modulator, probably by interaction with SlMADS-RIN [20].

In citrus, fruit ripening has been studied in several transcriptomic analyses providing a general view of the transcriptome evolution during maturation in Clementine mandarin [5], grapefruit [21], Ponkan mandarin [22] and sweet orange [23]. Relevant information about regulation of the different aspects of citrus fruit ripening was obtained from comparative studies of bud mutations (bud sports). These mutations are a consequence of genetic variation of somatic cells leading to the occurrence of phenotypic alteration in plants, that occur spontaneously in buds and limbs, and represent the main natural source of new cultivars [24]. The new phenotype is generally maintained by vegetative propagation by clonal techniques, leading to a new cultivar [25]. Bud mutant selection is the most common method for creating novel cultivars in Citrus and generally is carried out by the growers themselves, who detect branches of trees showing altered horticultural traits, such as maturity and flowering time or fruit characteristics [26].

The bud mutant and its original cultivar are excellent materials to identify and describe the molecular mechanisms involved in citrus fruit maturation [5]. Some mutants display changes in fruit color, like the ‘Tardivo’ mandarin, a late ripening mutant of the ‘Comune’ clementine and Mingliutianju, a late-ripening mutant of Chuntianju, that have been analyzed at the transcriptome level [27, 28]. More recently, RNA-Seq technique has been used for transcriptome comparative studies between wild sweet orange cultivars and several mutants: Hong Angliu orange that displays a red flesh phenotype [29]; and mutant oranges Fengwan [30], and Jincheng [31] that produce late ripening fruits. A similar analysis has been carried out in the late-maturing mandarin `Huawan Wuzishatangju’ and its original line `Wuzishatangju’ [32]. In all cases, the analysis of the transcriptome changes showed the large number of differentially expressed genes involved in the appearance of the new phenotypes, and that how these mutations affect many of the pathways previously described during fruit ripening of citrus fruits: cell wall biosynthesis, carbohydrate and citric acid metabolism, carotenoid metabolism, chlorophyll degradation, etc., that would be regulated by ABA, sucrose, jasmonic acid, and ethylene by interacting with each other [22, 23].

**Fig. 1:**
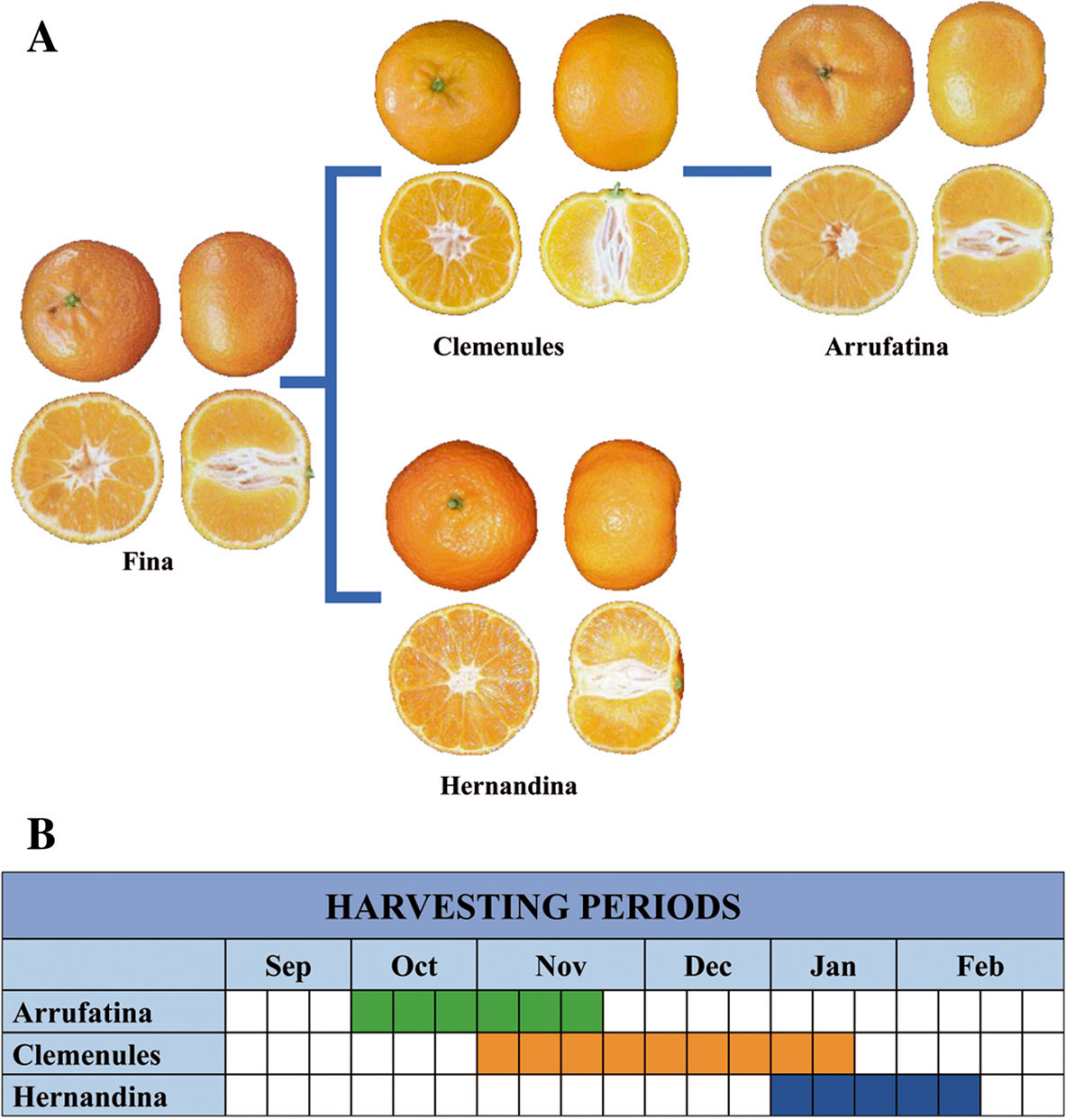
*Citrus clementina* cultivars used in this study. Hernandina and Clemenules are sport mutations derived from Fina, while Arrufatina is derived from Clemenules (**a**). The harvesting periods of the 3 cultivars classifies Clemenules as a mid-term cultivar, Arrufatina as an early one and Hernandina as a late cultivar (**b**)

In this work we analyze 3 sport mutations that belong to the Fina group of mandarins: Clemenules (CLE) and Hernandina (HER) are sport mutations derived from Fina, and Arrufatina (ARR) is a bud mutation originated from Clemenules (Fig. 1a). Clemenules, is considered a mid-term-ripening mandarin, its harvesting period comprising from November till mid-January, while Arrufatina, an early-maturing cultivar is collected at least 4 weeks in advance, starting in October, and Hernandina, a late-ripening cultivar, is harvested till February, 3 weeks later than Clementine. (Fig. 1b). Transcriptional analysis was carried out with RNA-Seq technology along the ripening period from September till December, in a time-course study of gene expression that was expected to shed light on the mechanisms controlling fruit maturity date. The study of these cultivars offers an opportunity to identify the mechanisms involved in the determination of harvesting date, that, considering the economic relevance of the precocity and lateness traits, will provide relevant information for marker assisted breeding of early- or late-maturing new clementine cultivars.

## Results

### Physiological characterization of the 3 cultivars shows the differences during ripening

**Fig. 2:**
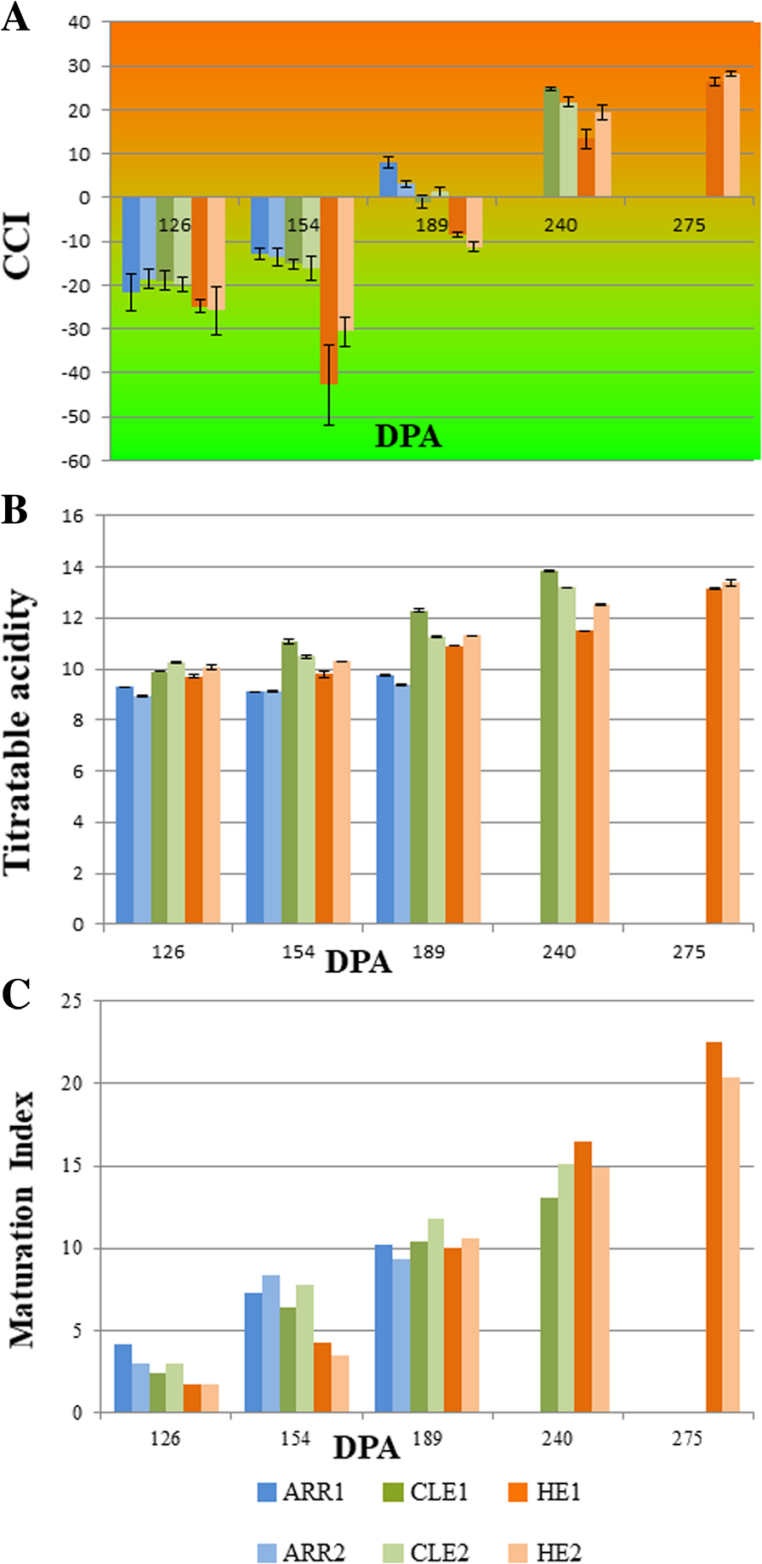
Physiological characterization of ripening. **a** Evolution of color during fruit ripening: the standard citrus color index CCI, based on Hunter L, a, b system [86], was used to measure color change (Y axis). Negative values are indicative of green color, while positive ones of orange color, 0 signals color break. It can be appreciated that at 189 DPA ARR peel has turned to orange while in HER is still green. **b** Evolution of titratable acidity during fruit ripening, measured as grams of citric acid per 100 ml of juice (Y axis). The bar graph shows that at 126 DPA ARR has half the acidity levels than HER, while at 189 DPA the acidity of the 3 cultivars is still similar. **c** Evolution of maturity index as the rate of BRIX and acidity. It is evident that at 126 and 154 DPA, MI is higher in ARR and lower in HE with respect the control CLE, which is in agreement with the early and late phenotypes that characterize ARR, HE, respectively. At 189 DPE MI was similar in the 3 cultivars. X axis represents days post anthesis (DPA) and error bars indicate SEM

Samples were collected at different ripening states: 126, 154, 189, 240 and 275 DPE (Table 1). The ripening state of each cultivar was determined by measuring titratable acidity [33] of the pulp and citrus color index (CCI) [34] of the fruit rind for all samples at the indicated dates (Fig. 2). The main difference in the external ripening, can be appreciated at 189 Days Post Anthesis (DPA), when ARR has clearly turned to orange color, while CLE hardly begins to de-greenish, and HER still has green fruits. CLE reaches similar CCI values an average of 10 days later than ARR and 23 days in advance to HER, as extrapolated from the graph (Fig. 2a).

**Table 1:** Samples analyzed with RNA-seq

Sample	Cultivar	COL. DATE	DPA	EBI code
CL126R1	CLEMENULES	3/09/13	126	ERS1069615
CL126R2	CLEMENULES	3/09/13	126	ERS1069616
AR126R1	ARRUFATINA	3/09/13	126	ERS1069633
AR126R2	ARRUFATINA	3/09/13	126	ERS1069634
HE126R1	HERNANDINA	3/09/13	126	ERS1069623
HE126R2	HERNANDINA	3/09/13	126	ERS1069624
CL154R1	CLEMENULES	1/10/13	154	ERS1069617
CL154R2	CLEMENULES	1/10/13	154	ERS1069618
AR154R1	ARRUFATINA	1/10/13	154	ERS1069611
AR154R2	ARRUFATINA	1/10/13	154	ERS1069612
HE154R1	HERNANDINA	1/10/13	154	ERS1069625
HE154R2	HERNANDINA	1/10/13	154	ERS1069626
CL189R1	CLEMENULES	5/11/13	189	ERS1069619
CL189R2	CLEMENULES	5/11/13	189	ERS1069620
AR189R1	ARRUFATINA	5/11/13	189	ERS1069613
AR189R2	ARRUFATINA	5/11/13	189	ERS1069614
HE189R1	HERNANDINA	5/11/13	189	ERS1069627
HE189R2	HERNANDINA	5/11/13	189	ERS1069628
CL240R1	CLEMENULES	26/12/13	240	ERS1069621
CL240R2	CLEMENULES	26/12/13	240	ERS1069622
HE240R1	HERNANDINA	26/12/13	240	ERS1069629
HE240R2	HERNANDINA	26/12/13	240	ERS1069630
HE275R1	HERNANDINA	30/01/14	275	ERS1069631
HE275R2	HERNANDINA	30/01/14	275	ERS1069632

The titratable acidity graph shows how, at 126 and 154 DPA, ARR displays half levels of acidity with respect to HER, showing the advancement or delay of the internal ripening process, respectively. CLE shows similar acidity values 10 days later than ARR and 20 days earlier than HER (extrapolated from graph), in a similar way than color change (Fig. 2b). At 189 DPA acidity is similar in the pulp of the fruit cultivar, which is in contrast with the differences in the CCI that the fruit rind still displays, evidencing the unpairing of the internal (pulp) and external (peel) maturation that takes place in citrus fruits and that has been previously reported [35, 36].

Maturity index (MI), a relation between the BRIX (sugar content) and acidity, is used an indicator of the internal ripening state. The different MI levels in the 3 cultivars are evident at 126 and 154 DPA, confirming that the early and late phenotypes affect internal maturation of the fruit (Fig. 2c). Unlike color change, at 189 DPA the 3 cultivars display similar MI, showing how internal and external ripening processes are not coupled.

### Overview of RNA-seq analysis

RNA-Seq was carried out as described in Experimental procedures section, and the results are summarized in Additional file 1. Total RNA was extracted separately from peel and pulp in order to optimize RNA extraction, as water content of these tissues is very different, so performing the extractions separately we were certain that equal amounts of total mRNA from both tissues were mixed and used for library construction.

Twenty-four pair-end libraries were constructed and sequenced with 75 bp reads. After quality trimming a total of 1.86 billion reads were obtained, and the average number of reads per sample was 77.6 million, accounting for 140.7 Gb of useful sequence.

Reads were mapped against the 27,837 transcripts of the citrus reference transcriptome [37], as described in Methods section. Overall, 1457.8 million reads mapped in pairs, 342.4 million reads mapped in broken pairs, while 59.6 million reads din not map. The reads mapped to exons were 666 million, whereas 62.8 million reads mapped to introns, with a total of 728.9 million reads mapped to genes. Considering the total size of the transcriptome (81.1 Mb), the coverage per transcript ranged from 18x to 35x, with an average of 25x; the average number of reads mapped per gene was 75,200. Detailed mapping results for each sample can be found at Additional file 1.

### Comparative time-course analysis of fruit ripening identifies clusters of genes with different expression patterns

In order to identify those genes that displayed differential expression patterns during fruit ripening, we used maSigPro [38] with the RNA-Seq data to perform a time-course analysis. The aim was to identify those genes that showed different expression patterns in the early- (ARR) or late-maturing (HER) phenotypes with respect the reference (CLE).

**Fig. 3:**
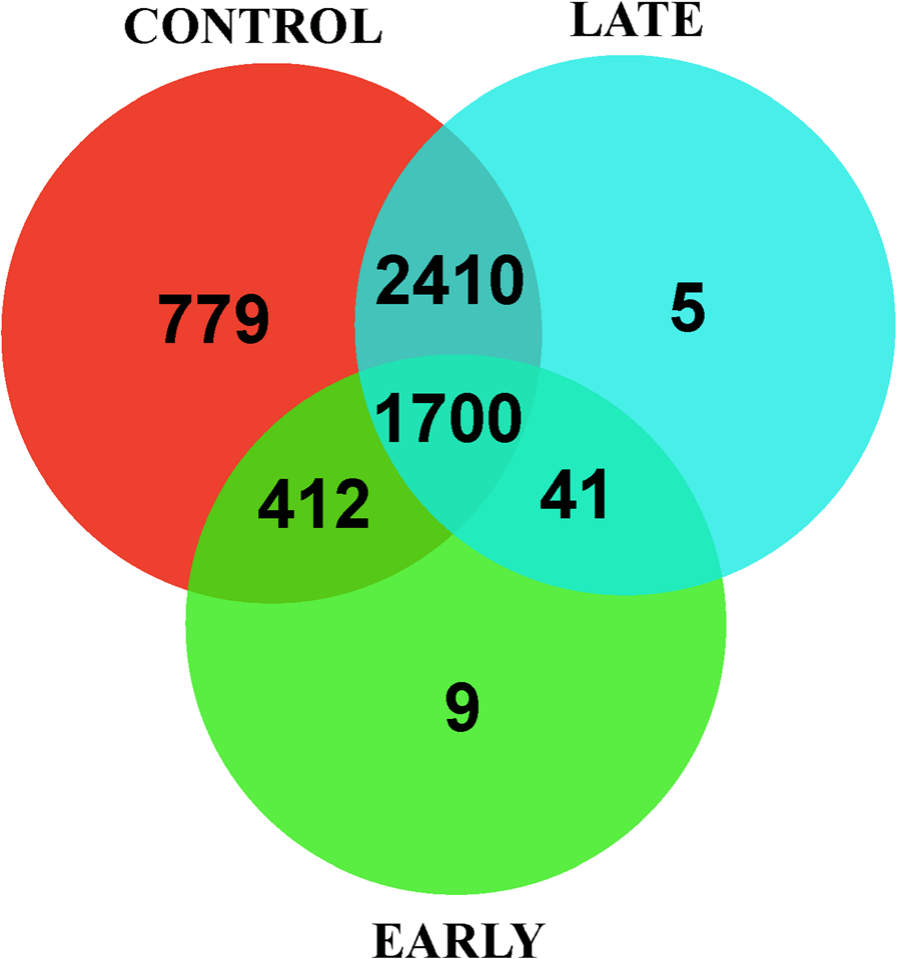
Venn diagram summarizing the results of maSigPro time course analysis. 5356 genes were differentially expressed that were grouped in Clusters Control, Early, Late and Early-Late depending on the differences in their expression patters during maturation processes in the 3 cultivars: ARR (early), CLE (control) and HER (late). 779 DEGs didn’t show different expression patterns, 412 DEGs presented differences between Early and Control, 2410 DEGs between Late and Control trends, and in 1700 DEGs the differences were detected among the three groups. Analyses were performed on samples with mixed mRNA from peel and pulp (see methods)

maSigPro selected 5356 differentially expressed genes (DEGs) with FDR = 0.05 and R2 = 0.7, that showed expression patterns that varied along the ripening process and were grouped in different clusters based on their expression profiles. Results are summarized in a Venn diagram in Fig. 3, and a list with all the genes and the clusters are provided in Additional file 2. Clusters were named as Control, Early, Late and Early-Late depending on the differences found among the 3 analyzed cultivars.

**Fig. 4:**
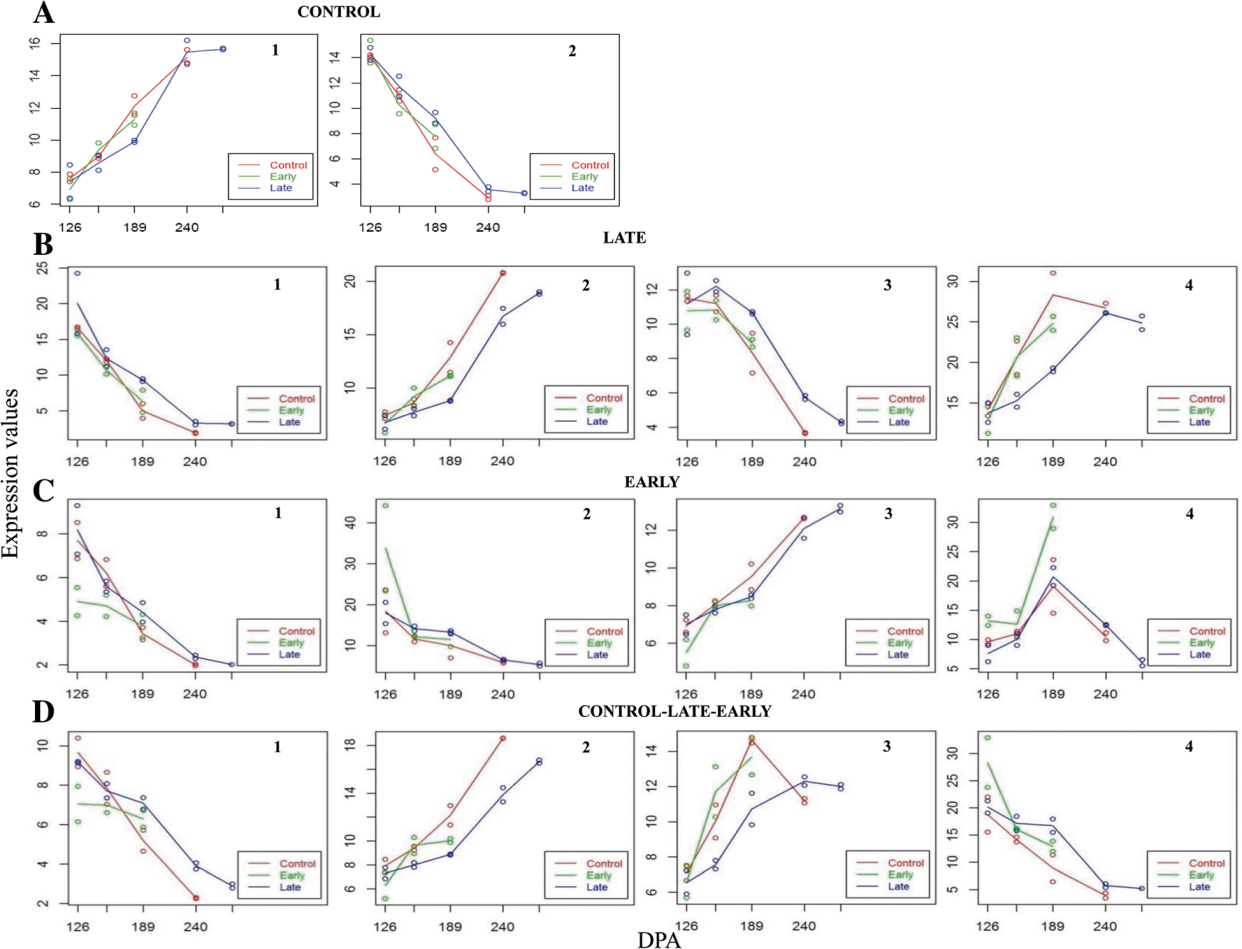
Gene expression plots. All the genes for which differences between the experimental groups were detected as significant by maSigPro time course analysis are shown. DEGs that didn’t show different expression patterns group in 2 clusters with increasing and diminishing expression level (**a**). 4 clusters of DEGs presented differences between Early and Control, with clusters 1 and 3 showing decreasing expression and 2 and 4 with the opposite trend (**b**). DEGs with differences between Late and Control also form 4 clusters, with clusters 1 and 2 increasing the expression with time, cluster 3 with a decreasing one and cluster 4 reaching a peak at 189 DPA (**c**). Finally, DEGs with differences detected among the three groups also display different expression patterns with clusters 1 and 4 showing decreasing trends, and clusters 2 and 3 with increasing ones (**d**)

Genes in Control clusters displayed expression patterns that were similar in the 3 cultivars, with ARR and HER showing no differences with respect CLE (Fig. 4a). 501 genes in cluster Control-1 increased significantly their expression during ripening, while 278 genes in cluster Control-2 displayed decreasing expression levels.

Late clusters contained 2410 genes that showed a different expression profile in HER, while CLE and ARR had similar patterns. 4 late clusters of 525, 956, 656, and 273 genes were grouped based on their common expression patterns, with clusters Late 1 and 3 presenting decreasing expression levels, while genes in clusters Late 2 and 4 showing increasing ones (Fig. 4b). The most remarkable feature common to the 4 clusters is the clear delay in gene expression of HER with respect CLE and ARR, that was estimated in 30 days as an average, with values ranging from 20 to 30 days, depending on the cluster and the date analyzed.

Four hundred twelve genes that presented differences between the early cultivar ARR, with respect CLE and HE were grouped in 4 Early cluster with 143, 76, 174, and 19 genes, based on their expression profiles (Fig. 4c). Gene expression in 2 of these clusters showed a clear advancement, as similar expression values could be found in ARR 28 days before CLE and HER as an average. These differences were higher at 126 DPE with some 42 days of precocity, and decreased over time, being only 14 days at 189 DPE.

Finally, a large set of 1700 genes showed different patterns in the 3 cultivars that were arranged in 4 Early-Late clusters, containing 689, 673, 161, and 177 genes (Fig. 4d). In the four clusters the delay on gene expression is evident for HER, while the advancement for ARR is not so apparent in 2 of them.

**Fig. 5:**
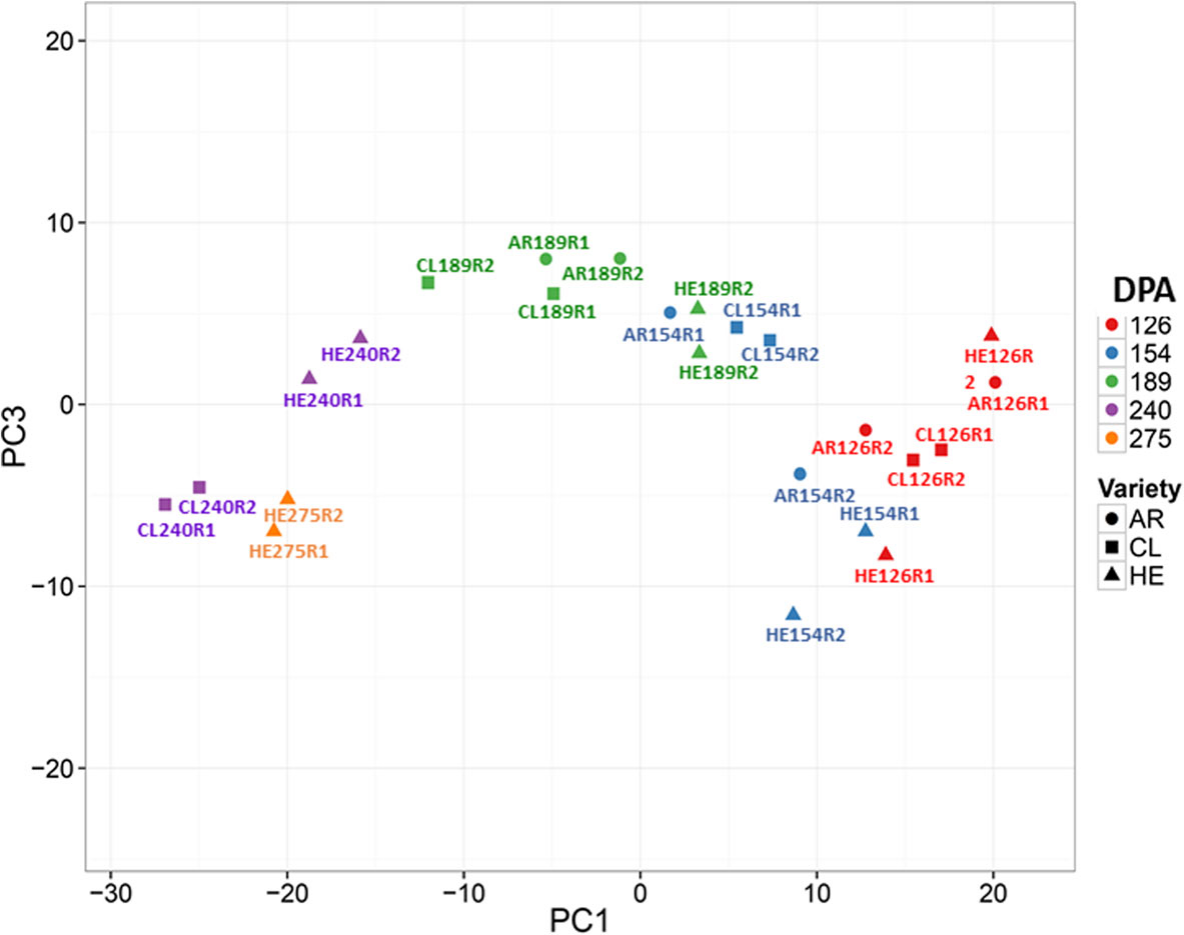
Principal components analysis. PCA, based on the RNA-Seq expression data of the whole transcriptome from the 24 samples analyzed. The samples don’t group by day collection or cultivar, showing the delay or the advancement of gene expression in HER and ARR

The shift in gene expression becomes apparent in the results PCA performed with all the samples with the expression values of 24 samples. The graph shows how HER at 154 is closer to 126 DPA samples, HER at 189 is clusters with 154 DPA samples and HER at 275 groups with CLE at 240 DPA. (Fig. 5).

### Functional annotation of the transcripts shows the main processes involved in ripening

Functional annotation of genes differentially expressed was carried out with BLAST2GO [39], and KEGG database [40] was used to identify those pathways involved in fruit ripening. Overall, 5600 genes were annotated with GO terms, and 1471 could be assigned to known metabolic pathways.

The most represented pathways (Table 2) include the Plant hormone signal transduction, the Oxidative phosphorylation, the Starch and sucrose metabolism, or the Glycolysis/gluconeogenesis ones. The most abundant GO terms (Additional file 3) include response to oxidation-reduction process, regulation of transcription, response to abscisic acid, sucrose metabolic process, transmembrane transport or pentose-phosphate shunt. Our analyses focused on the genes involved in color change and fruit acidity, the 2 traits measured in this work in all samples.

**Table 2:** Most abundant pathways in clusters

KEGG pathway	Early	Late	Early-Late	TOTAL
Plant hormone signal transduction	6	21	23	50
Oxidative phosphorylation	1	37	1	39
Starch and sucrose metabolism	4	16	17	37
Glycolysis / gluconeogenesis	3	20	10	33
Amino sugar and nucleotide sugar metabolism	0	19	13	32
Carbon fixation in photosynthetic organisms	4	13	9	26
Phenylpropanoid biosynthesis	0	12	12	24
Glycerolipid metabolism	1	10	12	23
Glyoxylate and dicarboxylate metabolism	2	19	2	23
Photosynthesis	3	17	3	23
Glycine, serine and threonine metabolism	2	12	8	22
Pyruvate metabolism	1	13	8	22
Porphyrin and chlorophyll metabolism	2	12	6	20
Purine metabolism	1	10	8	19
Pentose and glucuronate interconversions	2	10	6	18
Cysteine and methionine metabolism	1	9	8	18
Phenylalanine tyrosine and tryptophan biosynthesis	4	11	3	18
Glutathione metabolism	0	10	6	16
Citrate cycle (TCA cycle)	1	11	4	16
Glycerophospholipid metabolism	1	8	7	16
Pentose phosphate pathway	3	6	5	14
Lysine degradation	0	9	4	13
Galactose metabolism	2	6	5	13
Ascorbate and aldarate metabolism	0	8	4	12
Arginine and proline metabolism	0	8	4	12
Valine leucine and isoleucine degradation	0	10	2	12
Fructose and mannose metabolism	2	7	3	12
Alanine aspartate and glutamate metabolism	0	8	3	11
Fatty acid biosynthesis	1	6	4	11
Inositol phosphate metabolism	0	6	5	11
Terpenoid backbone biosynthesis	0	6	4	10
Carotenoid biosynthesis	1	4	3	8
Flavonoid biosynthesis	0	5	1	6
N-Glycan biosynthesis	1	2	3	6
Circadian rhythm	1	3	2	6

Change from the green color of the peel of immature fruits to the orange tints of the peel and pulp of mature fruits, implies degradation of chlorophyll [41], and carotenoids biosynthesis [42]. Development of pigmentation is a major feature during ripening of mandarins, and the time of color change determines greatly the classification as early- or late-maturing cultivars. Accordingly, genes involved in these processes were found in the clusters of DEGs identified in the time course analysis (Additional file 2). Regarding the chlorophyll degradation (Additional file 4), genes coding for chlorophyllase 1 (*Ciclev10021095*, acc n° XM_006441400), chlorophyllase 2 (*Ciclev10005453*, acc n° XM_006419948.2), accelerated cell death 2 (*Ciclev10026248*, acc n° XM_006425829 and *Ciclev10026041,* acc n° XM_006425830) were found in Late 2, 3 and 4, and Late-Early 1 clusters. As a general trend, from the 42 genes related to chlorophyll biosynthesis or degradation, those annotated with chlorophyll, chlorophyll a biosynthesis or chlorophyll cycle belonged to clusters that presented declining expression patterns, like Early 1, Late 1 and 3, and Late-Early 1 and 4. On the other hand, genes annotated as chlorophyll A degradation or chlorophyll catabolic process belonged to clusters with increasing expression levels, like Late 2 and 4, Early 3 and Late-Early 2 and 3. These data would be in agreement with the end of the synthesis and the start of degradation of chlorophylls during fruit maturation [43].

Several genes coding for the enzymes responsible of the synthesis of carotenoids [42], the pigments that provide the orange color of the mandarin, were also present in the clusters of DEGs Late 1 to 4 and Early-Late 1 and 2: carotene desaturase *Ciclev10002967* (acc n° XM_024183756), lycopene epsilon cyclase *Ciclev10008410* (acc n° XM_024178617), lycopene beta cyclase *Ciclev10028245* (acc n° XM_006424132), phytoene synthase *Ciclev10011841* (acc n° XM_006430334), phytoene synthase *Ciclev10015582* (acc n° XM_006445756), cytochrome P450 *Ciclev10011312* (acc n° XM_006428483) and *Ciclev10011420* (acc n° XM_006428462), carotene isomerase *Ciclev10020648* (acc n° XM_006443093), and carotene hydroxylase *Ciclev10005481* (acc n° XM_006421968) (Additional file 5). Especially relevant in these clusters is the delay of the gene expression in HER, which is in agreement with the observed phenotype of this cultivar, that changes color 2 weeks later than CLE.

Pulp acidity of citrus fruit (TA) is a key factor of fruit flavor quality that in citrus is correlated with the citric acid concentration. Citrate accumulates during the growing phase of the ripening and after reaching a peak when the fruit volume is about 50% of its final value, declines gradually as the fruit matures [44]. We found 14 genes involved in the synthesis, accumulation and catabolism of citric acid present in the clusters of DEGs produced in the time course analysis (Additional file 6). Half of the TCA-cycle genes identified were found in late clusters: oxoglutarate dehydrogenase *Ciclev10018656* (acc n° XM_006441600), aconitase 3 *Ciclev10014140* (acc n° XM_006447492), isocitrate dehydrogenase *Ciclev10014816* (acc n° XM_006446487) and *Ciclev10011936* (acc n° XM_006428693), malate dehydrogenase *Ciclev10028730* (acc n° XM_006422620) and *Ciclev10025945* (acc n° XM_006426114), succinate dehydrogenase *Ciclev10016181* (acc n° XM_006447142) and *Ciclev10025149* (acc n° XM_006425624). Only malate dehydrogenase *Ciclev10020378* (acc n° XM_006440542) was found in an early cluster (Early3), which can be related with the differences in acidity levels found in HE with respect CLE and ARR till 189 DPA (Fig. 2).

### Analysis of transcription factors expressed in Clementina fruits identifies genes that might play relevant roles during ripening

The results of the time-course study show how the early and late phenotypes correlate with a shift in the expression of genes involved in ripening, but don’t unvail the origin to the precocity or lateness of ripening in the analyzed cultivars. Therefore we extended further our analysis to study the regulation of maturation, and the transcription factors that might be responsible for the ARR and HER phenotypes.

In a first approach, we identified the transcription factors putatively involved in citrus fruit ripening. Thus, all the TFs present in the *C. clementina* proteome were obtained using the PlantTFcat database [45], which yielded a total of 3966 regulatory proteins. 580 of them belonged to the group of 5356 genes differentially expressed during ripening, which represents 14% of the total TFs found, and 11% of the differentially expressed genes (Additional file 7). Among this 593 TFs, the most abundant families were the Zinc finger with 117, WD40-like with 59, MYB with 35, and AP2-EREBP with 34 genes; besides, 8 MADS box proteins and 6 AUX-IAA responsive factors were also identified. The large number of TFs found is in agreement with the functional annotation results, with the GO term “regulation of transcription, DNA-templated” as the second most abundant, and show the tight genetic control to which fruit ripening is subjected.

In a complementary approach, a differential expression analysis of all the almost 4000 TFs obtained with PlantTFcat, plus some additional TFs previously identified by functional annotation, was performed, comparing the expression level of the genes in Clemenules fruits against that obtained for leaves, roots and phloem in a previous RNA-Seq study with samples from the same tree [37]. A total of 273 transcription factors were identified as differentially expressed in fruit with 3-fold change and FDR 0.05 as cutoff values. 40 TFs had been previously identified in the time course analysis, supporting the idea that these factors can display relevant roles during fruit ripening and, some of them, could be responsible of the early or late phenotypes of ARR and HE. (Additional file 8).

The most abundant families of TFs belonged to the C2H2, AP2-EREBP, MYB-HB-like, WD40-like, PHD, MADS-MIKC, bHLH and NAM/NAC families.

**Fig. 6:**
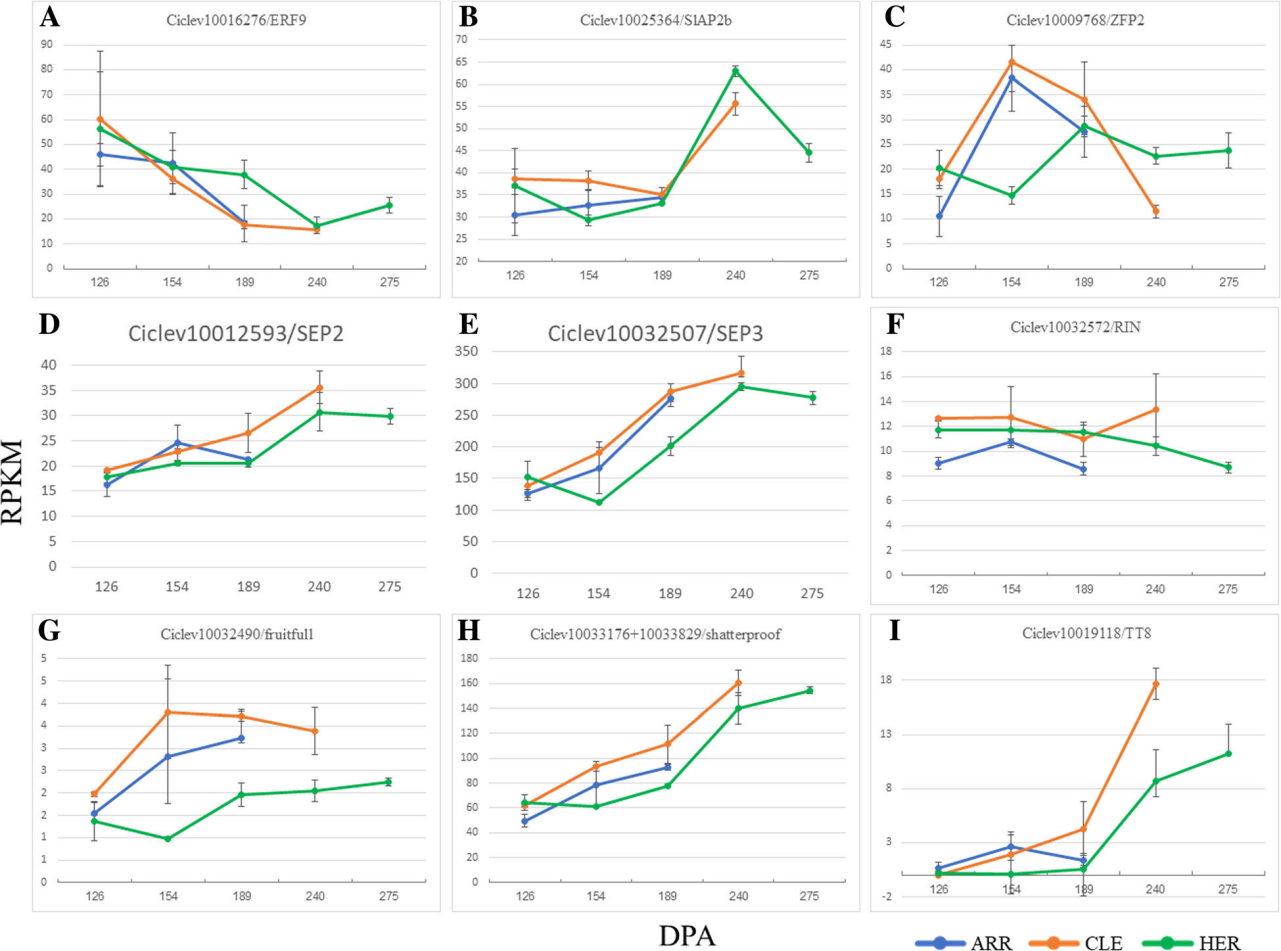
Expression patterns, of several transcription factors that are differentially expressed in clementine fruits and show relevant roles in other fruit crops. *Ciclev10016276* (**a**) and *Ciclev10025364* (**b**) belong to the AP2-ERF family; *Ciclev10009768* (**c**) is a C2H2 TF, similar to SlZFP2 from tomato; *Ciclev10012593* (**d**) codes for a SEP-like MADS box protein; *Ciclev10032507* (**e**), is a SEP3/AGL9 MADS box protein; *Ciclev10032572* (**f**) is SEP 4/AGL3 like gene, close to RIN; *Ciclev10032490* (**g**) is strongly related to AP1 1/Fruitful genes; *Ciclev10033829* (**h**) is similar to SHATERPROOF protein; *Ciclev10019118* (**i**) is a BHLH similar to Transparent Testa 8 protein. In all cases X axis represents days post anthesis (DPA) and Y axis RPKM expression values based on RNA-Seq counts obtained from libraries with a mixture of total RNA from pulp and peel, with error bars representing SEM values

Detailed annotation of the citrus TFs, based on BLASTP searches and phylogenetic analyses, showed that several of them were closely related to TFs with relevant roles in ripening control in citrus and other species, and their expression patterns were analyzed in detail (Fig. 6). 27 Apetala 2-ethylene responsive factors (AP2-ERF) were identified with Ciclev10016276 (acc n° XM_024191939, Fig. 6a), showing high similarity to ethylene response factor 3, LeERF3b, from tomato [46]. Also, Ciclev10025364 (acc n° XM_00642588), was very similar to SlAP2a, a tomato APETALA2/ERF gene [47] (Fig. 6b). Ciclev10009768 (acc n° XM_006453331, Fig. 6c), belongs to the C2H2 zinc-finger family, that was the most abundant in our analysis with 44 members, and was very similar to SlZFP2 from *Solanum lycopersicum* [48].

Similarity and phylogenetic analysis of the eleven proteins of the MADs family differentially expressed in citrus fruit, showed that five of them were the probable homologs of proteins with crucial roles in ripening in other species. Ciclev10012593 (acc n° XM_006430886, Fig. 6d), displays significant similarity with the developmental proteins SEPALLATA 1 and 2, while Ciclev10032507 (acc n° XM_006437932, Fig. 6e), was strongly similar to SEPALLATA 3/AGL9 proteins [49]. Ciclev10032572 (acc n° XM_006437815, Fig. 6f) clustered with SEPALLATA 4/AGL3 like proteins, [50], and Ciclev10032490 (acc n° XM_006437813, Fig. 6g) was strongly related to APETALA 1/Fruitful-like proteins [51].

Ciclev10033176 and Ciclev10033829 (acc n° XM_006437142 and XM_00643714 Fig. 6h) were very similar to SHATERPROOF-like protein [16]. Notably, Ciclev10033829 was similar to the 5′ end of the SHAT-like proteins and missed the 3′ end, that was the only part of the protein present in Ciclev10033176 (acc n° XM_006437142). A detailed analysis of the BAM file resulting from RNA-Seq analysis using the IGV genome browser, showed that both genes were part of the same transcript, and that the presence of a large intron of more than 6 kb had caused them to be annotated as different genes (Additional file 9). The resulting full-length protein was identical to proteins XP_024956880.1 and AVI01414.1 from *Citrus sinensis* and showed strong similarity to SHP 1 and 2 proteins from Arabidopsis, confirming our predicted protein based on RNA-Seq data.

Finally, we found that Ciclev10019118 (acc n° XM_006444030 Fig. 6i), a basic helix-loop-helix protein with high similarity to the Anthocyaninless protein from tomat [52].

Thus, the analysis of the expression pattern of these genes during ripening suggests that, like their homologs in other species, they might play relevant roles in the control of citrus fruit ripening. However, their expression patterns were not consistent with the late- or early-maturation phenotypes observed in ARR and HER.

### The gene *Ciclev10021357*, coding for a MADS box protein, could be related to the ARR and HER phenotypes

**Fig. 7:**
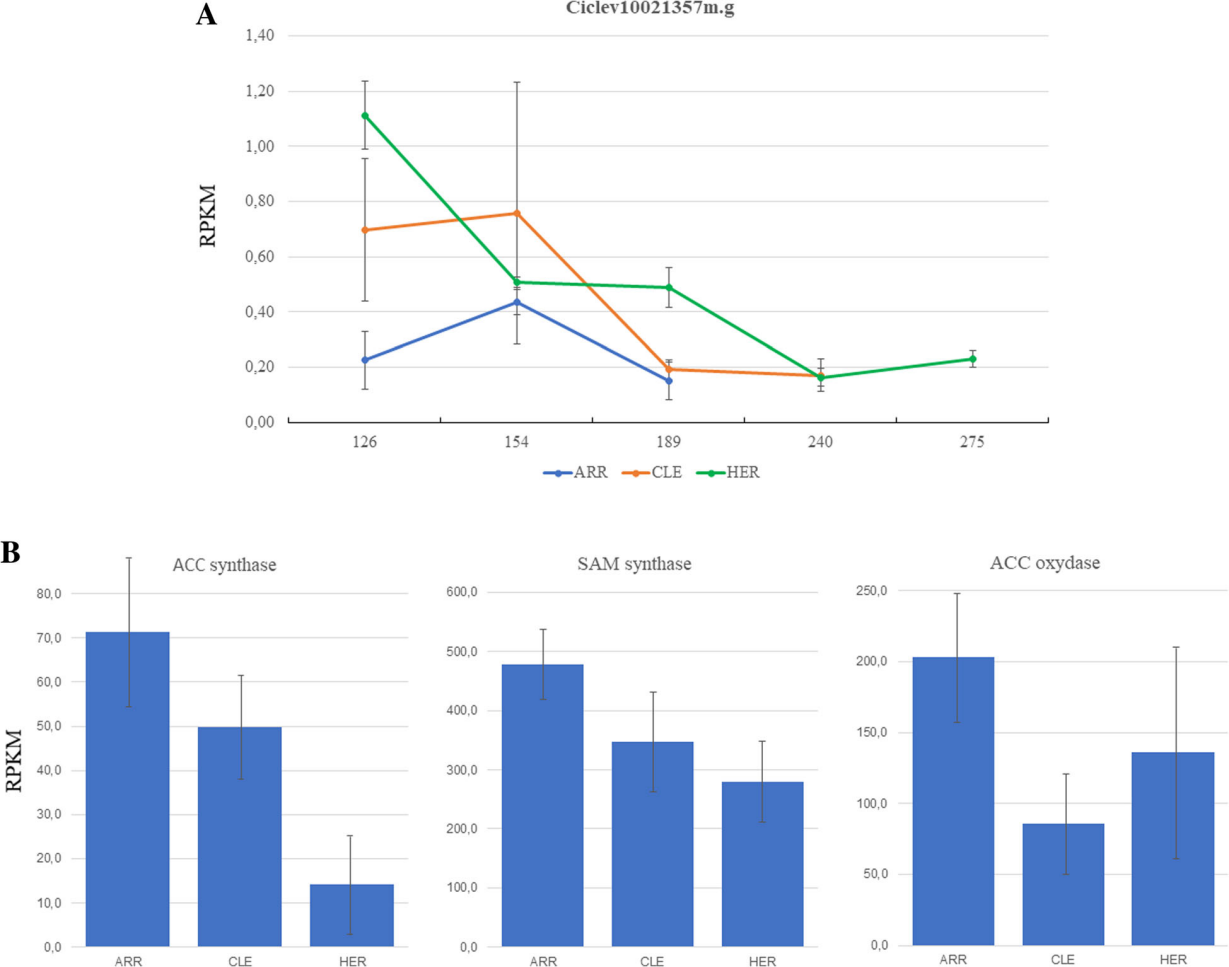
**a** Expression profile of *Ciclev10021357* during fruit ripening in ARR, LE and HER based on RNA-Seq counts obtained from libraries with a mixture of total RNA from pulp and peel. At 126 DPA expression is 5 times higher in HER than in ARR. **b** Bar charts showing the expression levels in RPKM of the genes responsible of ethylene synthesis, that are consistently higher in ARR when compared to CLE or HER, indicating higher levels of ethylene production. Error bars represent SEM values

**Fig. 8:**
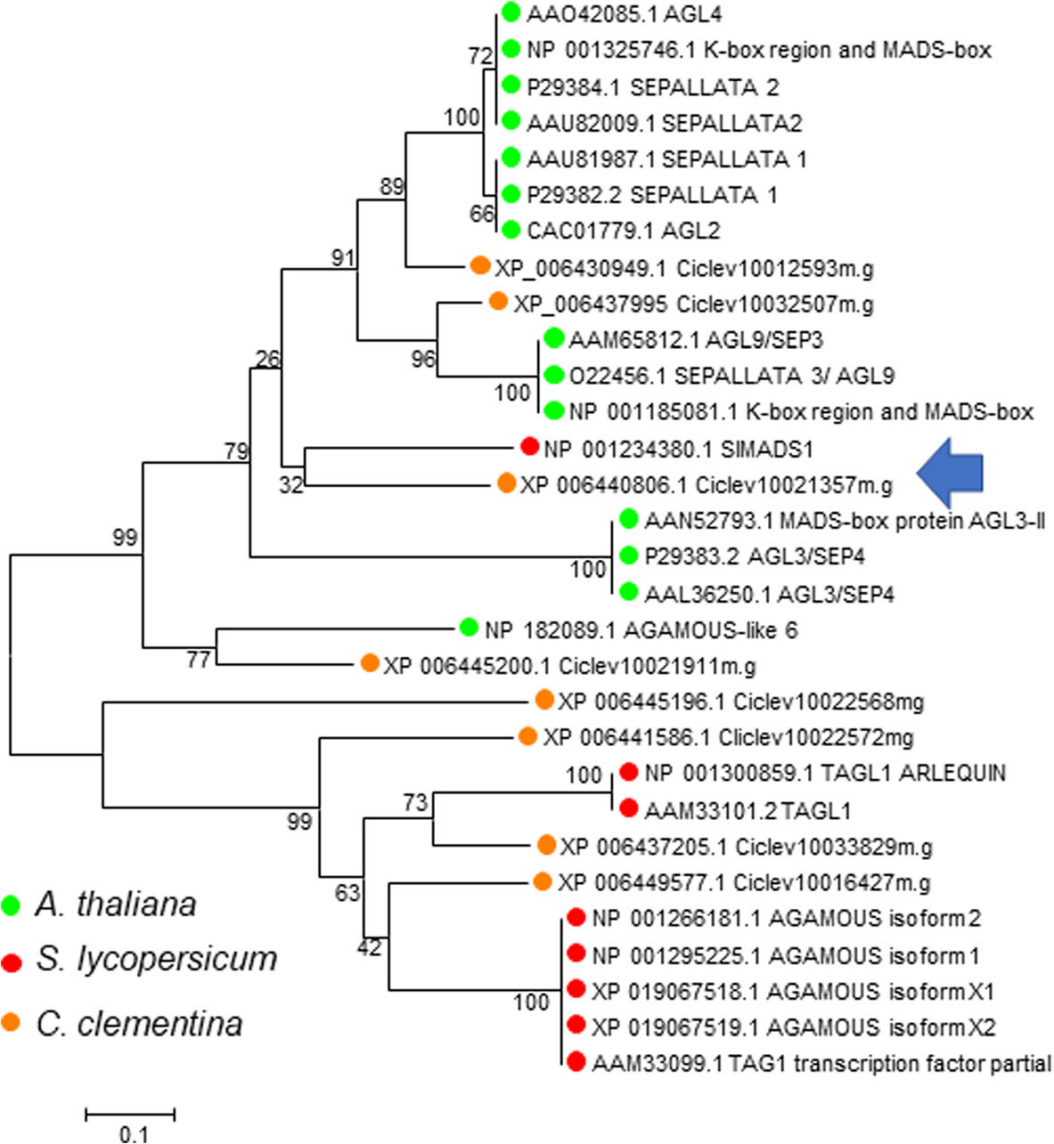
Phylogenetic tree with MADs box proteins from *Arabidopsis thaliana*, tomato and clementine. The evolutionary history was inferred using the Neighbor-Joining method. The percentage of replicate trees in which the associated taxa clustered together in the bootstrap test (500 replicates) are shown next to the branches. The tree is drawn to scale, with branch lengths in the same units as those of the evolutionary distances used to infer the phylogenetic tree. The evolutionary distances were computed using the Poisson correction method and are in the units of the number of amino acid substitutions per site. The close relationship of Ciclev10021357m.g and SlMADS1 can be appreciated (arrow)

Thereafter we searched for TFs with gene expression patterns correlating with the ripening phenotype of the three cultivars. Interestingly, there was a gene, *Ciclev10021357* (acc n° XM_024189910), that at 126 DPE showed and expression pattern that could be related with the early and late phenotypes. Although the expression levels are low, *Ciclev10021357* is differentially expressed in fruit, and at 126 DPA, the transcript levels are clearly lower in the early cultivar ARR and higher in the late cultivar HER, with respect the mid-season CLE (Fig. 7a). Ciclev10021357 protein belongs to the MADs box family of transcription factors and is the closest clementine relative of the tomato protein SlMADS1, as it can be appreciated in the phylogenetic tree on Fig. 8, were several MADs box proteins from Arabidopsis, tomato and clementine were analyzed. SlMADS1 is expressed in fruits; and its expression level decreased sharply in accordance with fruit ripening [20], a similar expression pattern is displayed by *Ciclev10021357*, as, after a small peak, its expression reduced to reach minimum levels at 189 DPE in ARR and CLE, and 240 DPE in HE.

In tomato, expression levels of the genes involved in ethylene biosynthesis, *ACC synthase* and *ACC oxidas*e were enhanced in plants with *SlMADS1* silenced, and fruits from silenced plants showed approximately 2- to 4-fold increases in ethylene production compared with the wild type [20]. To check the expression level of *ACC synthase*, *ACC oxidase*, plus *SAM synthase* transcripts in our samples, we identified in the reference genome of *C. clementina*, the genes coding for these proteins. We found 7 genes annotated with ACC synthase (EC:4.4.1.14), 3 with SAM synthase (EC:2.5.1.6) and 4 with ACC oxidase (EC:1.14.17.4) activities, however, only 1 *ACC synthase* (*Ciclev10019920*, acc n°), 1 *ACC oxidase* (*Ciclev10015962*, acc n°) and 2 *SAM synthase* (*Ciclev10011912*, acc n° *and Ciclev10020504*, acc n°) genes had relevant expression levels in fruits and were present in the clusters generated in the time course analysis. The RNA-Seq analysis showed that the expression levels for these genes was higher in ARR and lower in HER with respect CLE (Fig. 7b). The accumulation of carotenoids and the expression of *PHYTOENE SYNTHASE1* were also enhanced SlMADS1-silenced tomatoes, although we could not find such increase in ARR [20].

### qRT-PCR analysis confirms the results of RNA-Seq

In order to validate the results of the RNA-Seq study, 5 genes were selected for qRT-PCR analysis, based on its relevance for this study. Total RNA extracted from peel used in the RNA-Seq was also utilized in these experiments, that were carried out as described in Methods.

The genes and the primers used for PCR are shown in Additional file 8. *Ciclev10021357*, the SlMADS1 homolog, *Ciclev10032572* a close citrus relative to RIN, *Ciclev10020575* (acc n° XM_024188340) significantly similar to Agamous like AGL65 from *Arabidopsis* and to *Musa acuminata MaMADS7* proteins [49], and *Ciclev10021100* (acc n° XM_006440759), similar to a banana MaDof23 portein [53]. *Ciclev10020575* and *Ciclev10021100*, as *Ciclev10021357*, are located in the deleted region on chromosome 3 in ARR, and both of them showed lower levels of expression in ARR, but unlike *Ciclev10021357*, their expression patterns were not consistent with the observed ARR or HER phenotypes. *Ciclev10019920*, coding for an ACC synthase protein was also included, in order to confirm the differences of expression found in the RNA-Seq analysis.

**Fig. 9:**
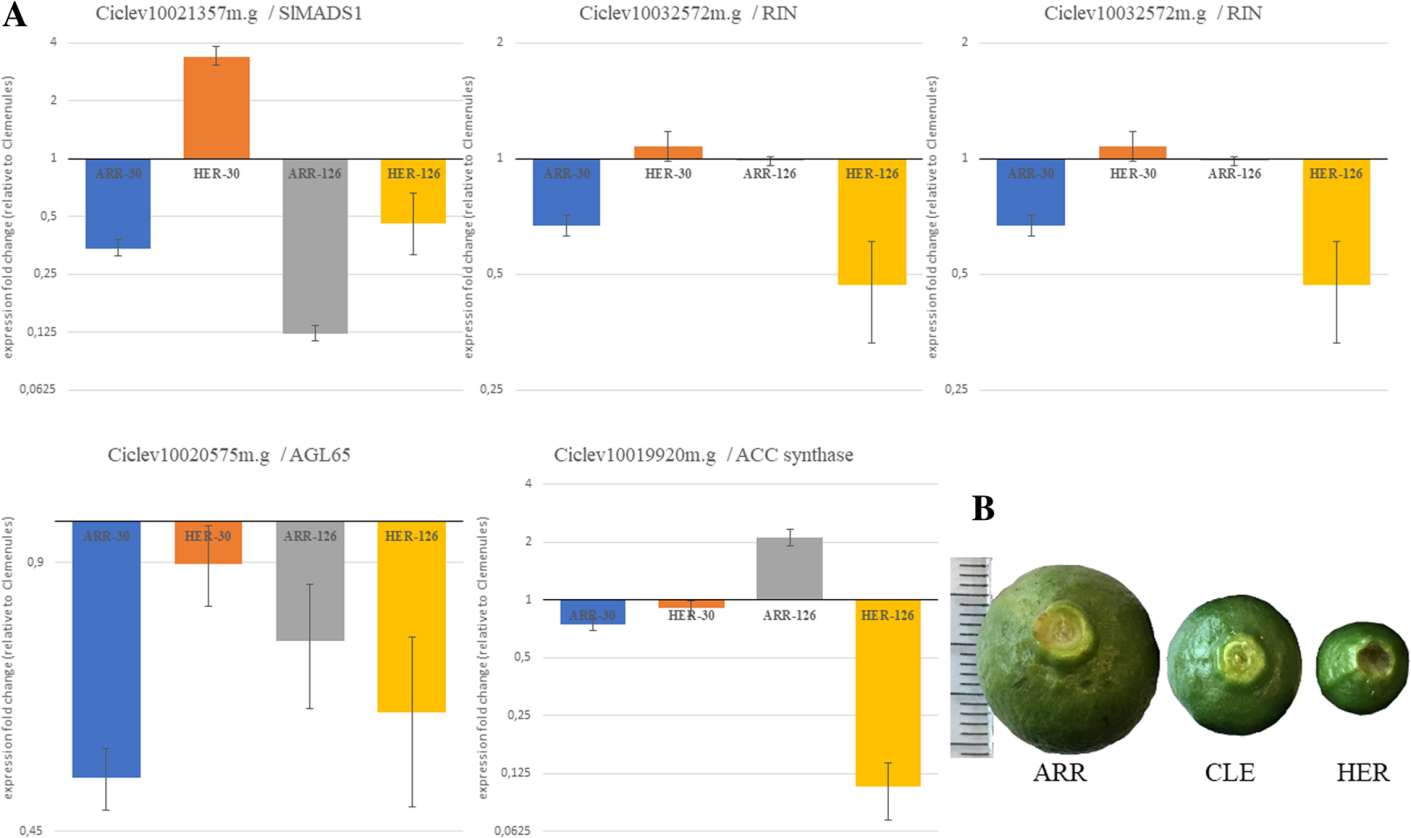
qRT-PCR experiments. **a** Results of the qRT-PCR experiments, shown as expression fold change of ARR and HER relative to CLE at 30 and 126 DPA. Original RNA from peel at 126 DPA and newly extracted from fruitlets at 30 DPA were used. The quantitative analysis confirms the RNA-Seq data for *Ciclev10021357*, the SlMADS1 homolog, *Ciclev10032572*, *Ciclev10021100, Ciclev10020575* and *Ciclev10019920*. **b** Image of ARR, CLE and HER fruitlets at 30 DPA, where the differences in size can be appreciated, the rule on the left side shows mm. Error bars represent SEM values

The expression fold change relative to Clemenules of the peel ARR and HER samples at 126 DPE, obtained by qRT-PCR, resembled the results obtained in the RNA-Seq analysis (Fig. 9a). The expression of *Ciclev10021357* is lower in ARR compared to CLE; although not higher in HER, yet the big error associated to this result can be misleading. *Ciclev10020575* and *Ciclev10021100*, the other genes located at the deletion on chromosome 3 in ARR show evident lower levels of expression in this cultivar, and similar to CLE in HER, which is in agreement with the RNA-Seq data. *Ciclev10032572*, the RIN-like gene, a promoter of ripening, show much lower expression in HER, while the levels in ARR are similar to those in CLE. Finally, the expression pattern for *Ciclev10019920*/Acc Synthase gene was also confirmed, with higher expression in ARR and lower in HER, with respect CLE.

In an effort to further investigate the genetic mechanisms involved in the ARR and HER phenotypes we analyzed with qRT-PCR the expression of the selected genes at 30 DPA, corresponding to ripening Stage I, characterized by slow fruit growth rates but high cell division. a high increase in thickness of the fruit rind and the beginning of the differentiation and growth of pulp vesicles [54]. At this stage there are evident differences in fruit size between the 3 cultivars, as shown by the average diameter of the fruitlets: 19 mm for ARR, 11 mm for HER and 15 mm for CLE, the control cultivar (Fig. 9b).

Results of the qRT-PCR of these 30 DPA samples are shown in Fig. 9a, in parallel to the data from the 126 DPA to facilitate comparison. The expression level of *Ciclev10021357* is in agreement with previous results, with lower levels in ARR and higher in HER when compared to CLE. The other genes analyzed showed similar patterns at both dates, except for the ACC Synthase gene, *Ciclev10019920*, that at 30 DPA doesn’t show clear differences in the expression levels, while at 260 DPA are clearly higher in ARR.

### A hemizygous deletion on chromosome 3 comprising Ciclev10021357 gene is responsible of its lower expression level in ARR

**Fig. 10:**
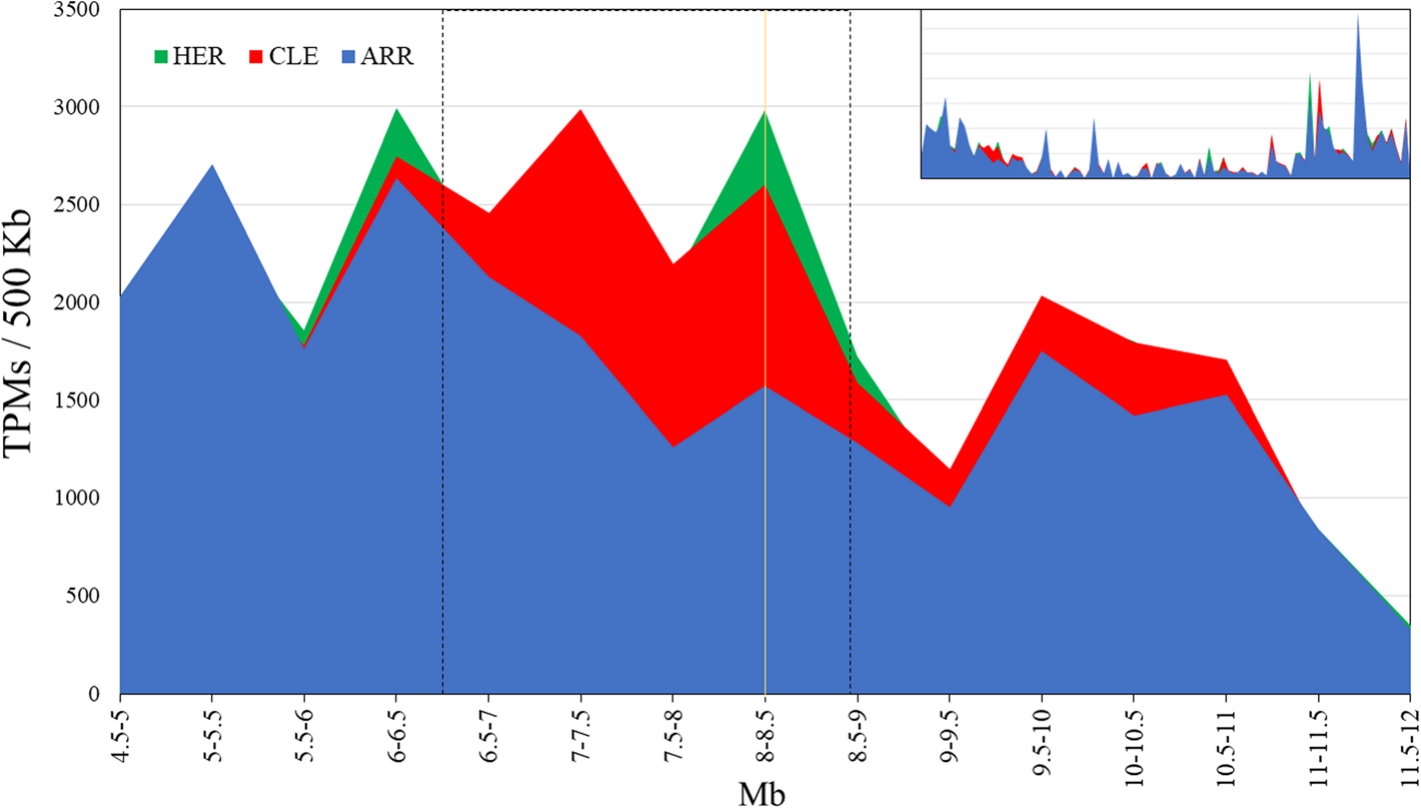
Global gene expression on the hemizygotic deletion. The area chart shows the expression level in ARR, CLE and HER in a region of chromosome 3 spanning from 4.5 Mb to 12 Mb, that includes the 2 Mb deletion in ARR. Expression is shown as total TPMs per 500 Kb. The small window on the upper right side shows the expression profile of the whole chromosome. The reduction of gene expression in the ARR hemizygous deleted region can be easily observed

As it has been described previously, ARR is a somatic sport mutation of CLE, characterized for its fruit precocity. Genomic analysis identified a hemizygotic 2 Mb deletion on chromosome 3 in ARR, spanning from positions 6.78 to 8.68 Mb, that is not present in CLE or HER [55]. *Ciclev10021357* is located between 8.45 and 8.46 Mb, inside the ARR deletion, that caused the gene to be in half dose, that can explain its lower level of expression in ARR. To confirm this idea, we analyzed the overall expression of the 225 genes present in the deleted region on chromosome 3, as well of those in the contiguous regions, comparing ARR with respect CLE and HE. Figure 10 shows the number of TPMs per 500 Kb, in a region of chromosome 3 spanning 7.5 Mb, from 4.5 to 12 Mb, including the hemizygotic deleted region and the adjacent areas. It can be observed that expression levels are evidently lower in ARR with respect CLE and HER in the deleted region, as it could be expected because the half genic dose present in the deletion. Therefore, the lower levels of expression of *Ciclev10021357* might be caused by the deletion of one copy of the gene.

## Discussion

The Physiological characterization of the 3 cultivars showed the differences during ripening, with a clear advancement in ARR and a delay in HER with respect the control cultivar CLE. The differences found for the acidity and CCI changes are in agreement with the unpairing of the internal (pulp) and external (peel) maturation that takes place in citrus fruits and that has been previously reported [35, 36]. It is important to notice that no major differences in flowering time have been ever reported for these 3 cultivars, so the phenotype observed can’t be due to advance/delay in flowering time, but has to be related to differences in ripening regulatory factors.

For preparation of RNA-Seq libraries, RNAs from peel and pulp were mixed at equal amounts, and, although this could affect the study of the unpaired internal and external maturation in citrus, missing some differences between maturation process in each tissue, it allowed us to analyze the 48 samples with the construction of 24 sequencing libraries. Considering that the main objective of our work was finding the differences between the early and late cultivars with respect the control, rather than the study of the evolution of the transcriptome during maturation itself, we would be able to study genes from both tissues included in the library and its evolution with fruit ripening.

Comparative Time-course analysis of fruit ripening identified clusters of genes with different expression pattern in the early- (ARR) or late-maturing (HER) phenotypes with respect the reference (CLE). This analysis shows that there is a clear delay in gene expression of HER, that was estimated in 30 days, while there is an advancement in ARR with an average 28 days. To our knowledge this is the first time-course study performed on citrus fruits, as in other works comparison of gene expression between late-maturing mutants and wild oranges showed differentially expressed genes just at one ripening state [29, 31, 32].

Both, the functional annotation of genes differentially expressed and the study of the most represented pathways, show that most of genes displaying differential expression patterns in HER and ARR cultivars are involved in the main processes taking place during ripening: to acid and sugar content, hormone signaling, cell wall remodeling, as well as pigment degradation and accumulation, which is in coincidence with previous RNA-Seq works [23, 29, 31, 32].

Therefore, the time course study performed in this work provides a landscape of the gene expression during ripening in early- and late-maturing cultivars, confirming previous results, and showing how the early or late phenotypes correspond with and advancement or delay of the genes that are involved in ripening and control different processes like degradation and synthesis of pigments, sugar accumulation, acid diminution, or size increment.

However, these results, although interesting, are just a consequence of the early- or late-ripening phenotypes and aren’t able to explain the ultimate causes that lead to the precocity or lateness of ripening in the analyzed cultivars, so it was necessary to analyze the transcription factors expressed in Clementina fruits that might play relevant roles during ripening. This analysis produced a large number of TFs previously described, which is in agreement with the results obtained for sweet orange in a similar study of late-ripening mutant Fengwan (*C. sinensis* L. Osbeck) its wild-type counterpart [56].

Several TFs were similar to proteins with relevant roles in ripening control in citrus and other species. The large amount of AP2-ERF factors identified could be expected considering the relevant role of the ethylene hormone in fruit ripening [57], that in citrus is especially relevant in the degreening and color change of the peel [58, 59]. Among these factors, LeERF3b, from tomato, with sequence similarity to the repressor class II of the ERF family, is proposed to play a repressor function in ethylene responses [46], while SlAP2a acts as a negative regulator of fruit ripening [47]. The C2H2 zinc-finger protein, SlZFP2, has been shown to negatively regulate abscisic acid biosynthesis and fruit ripening in tomato [48].

The MAD S box proteins SEPALLATA 1 and 2, initially characterized as some of the most relevant genes for flower differentiation, have been also shown as promoters of fruit ripening in strawberry [60] and peach [61]. Similarly, SEPALLATA 3/AGL9 protein from pepper affects fruit ripening both in ethylene-dependent and ethylene-independent aspects [62], and in banana plays an important role in initiating endogenous ethylene biosynthesis and fruit ripening [49]. The SEPALLATA 4/AGL3 like family includes the RIN protein from tomato, that has long been believed to function as a major inductor of ripening, although recent evidences contradicting this concept indicate that RIN is not required for the initiation of ripening and rin is not a null mutation, but rather is a gain-of-function mutation that produces a protein that actively represses ripening [50]. The APETALA 1/Fruitful-like proteins have been shown to regulate fruit ripening via ethylene biosynthesis in tomato [51]. SHATTERPROOF-like proteins have been described to control ripening in non-climacteric strawberries [63], and to regulate both fleshy fruit expansion and the ripening process in tomato [16]. Finally, the b-HLH p Anthocyaninless protein, involved in anthocyanin biosynthesis, is developmentally regulated and induced by low temperatures in tomato [64], and the homolog gene in peas has been found to determine anthocyanin pigmentation in pea, a character that was used by Gregor Mendel 150 years ago in his study of inheritance [52].

Thus, the analysis of the expression pattern of these genes during ripening suggests that, like their homologs in other species, they might play relevant roles in the control of citrus fruit ripening. In several of these genes like *Ciclev10012593*, *Ciclev10032507*, *Ciclev10032490* and *Ciclev10019118*, and even considering the error bars, a delay trend can be observed in the expression in HER with respect CLE and ARR. However, their expression patterns were not consistent with the late- or early-maturation phenotypes observed in ARR and HER.

The search for TFs with gene expression patterns correlating with the ripening phenotype of the three cultivars allowed the identification of the MADS box protein *Ciclev10021357*, that showed a clearly lower expression level in the early cultivar and higher in the late cultivar. Its closest tomato relative, SlMADS1, acts as a negative regulator of fruit ripening: RNAi silencing experiments targeting SlMADS1 produced shorter ripening time of tomato fruit, with enhancement of carotenoid and ethylene biosynthetic genes [20].

The expression levels at 126 DPE of *Ciclev1002135*7, and the role as a repressive modulator of ripening of SlMADS1, suggest a role for this TF in ARR and HE phenotypes. In ARR, with the lowest levels of *Ciclev1002135*7 expression, the repression of ripening would be diminished, and therefore there could be an advancement of the process with respect CLE. On the contrary, higher levels of *Ciclev1002135*7 transcripts in HER would increase inhibition and thus, could cause the ripening to be delayed.

Accordingly, RNA-Seq analysis showed that the expression levels of the genes involved in ethylene biosynthesis, *ACC synthase* and *ACC oxidas*e, that were enhanced in plants with *SlMADS1* silenced, [20], were higher in ARR and lower in HER with respect CLE, suggesting a possible effect of the downregulation of *Ciclev10021357* in the precocious ripening.

Unlike tomato, citrus fruits are classically considered as non-climacteric, due to the virtual absence of an increase in ethylene production and respiration rate during ripening, but there is evidence for the involvement of ethylene in the expression of specific RNAs during maturation of the orange [65], and application of exogenous ethylene accelerates color changes in the peel of fruits of most Citrus species and cultivars [59]. The analysis of the structure and transcriptional regulation of two climacteric (tomato and apple), and two non-climacteric (grape and citrus) fruits, indicated that both climacteric and non-climacteric fruits share many aspects of ethylene perception and signaling during fleshy fruit ripening, that puts into question the classic distinction between climacteric and non-climacteric patterns of ripening [66]. *CaMADS-RIN*, a SEP-like MADS non-climacteric fruit gene from pepper, close to SlMADS1, is able to regulate fruit ripening and ethylene biosynthesis in a climacteric tomato fruit, suggesting that CaMADS-RIN affects fruit ripening both in ethylene-dependent and ethylene-independent aspects, which provides evidence of the role of SEP genes in ripening of non-climacteric fruits [62].

The involvement of ethylene in different aspects of ripening in citrus fruits, specially color change, has been highlighted in the ‘Tardivo’ mandarin, a mutant of ‘Comune’ Clementine with a delay in peel degreening and coloration, that shows an altered sensitivity of the peel to ethylene-induced physiological and molecular responses, including fruit degreening and coloration processes [67]. Several ethylene responsive factors (ERFs) have been shown to affect internal ripening of citrus fruits: CitERF13, that regulates citric acid accumulation [68]; CitERF, that shows a strong expression in peel as well as in pulp during fruit ripening correlating with sugar content in the latter, indicating it might be a subject to the induction of sugar [69]. Finally, it has been shown that, upon harvest, fruitlets exhibited a climacteric-like rise in ethylene production, preceded by induction of the ACC synthase 1, ACC oxidase 1 and the ethylene receptor ERS1 genes, thus suggesting the existence of a system II-like pathway of ethylene production, that was thought to be exclusive of climacteric fruits [70].

Moreover, the fact that all the TFs with crucial roles in the promotion of the maturation process, mostly described in climacteric fruits, were differentially expressed in the non-climacteric citrus fruits would support the idea that the classic distinction between climacteric and non-climacteric patterns of ripening should be reviewed, as all the evidences point to a ubiquitous role of ethylene in fruit ripening [71].

qRT-PCR analysis confirms the results of RNA-Seq for all the genes selected. This is especially relevant for *Ciclev10021357*, the SlMADS1 homolog, as it supports its possible involvement of the early and late phenotypes of ARR and HER, as discussed above. We also chose other TFs with relevant roles in the ripening regulation: RIN acts as a promoter of maturation in tomato [50], *MaMADS7* plays an important role in initiating endogenous ethylene biosynthesis and fruit ripening in banana fruits [49], and the Dof transcription factor MaDof23, acts as a repressor and interacts with MaERF9 in regulating ripening-related genes [53]. The 3 genes located in the deleted region on chromosome 3 in ARR, showed lower levels of expression in this cultivar, but only *Ciclev10021357* expression was consistent with the observed phenotypes. The low expression level of the RIN-like gene, a promoter of ripening, could be expected in HER, where maturation is delayed. Finally, the expression pattern the Acc Synthase gene would, once again, reflect the early and late phenotypes of ARR and HER, and would support a relevant role of ethylene in the control of fruit ripening in citrus.

The qRT-PCR study was also performed on samples at 30 DPA, when the fruitlets already display evident differences ins size. As the final size of the 3 cultivars is very similar, the differences found at this stage must be caused by the advancement or delay in the ripening process, which matches with the larger fruits found in the early cultivar ARR in contrast with the smaller fruitlets from HER, the late cultivar.

Results of the qRT-PCR from samples at 30 DPA confirm again the expression pattern of *Ciclev10021357*, adding more evidences to its possible role in the control of ripening, and suggests an early role for this gene in the process. The differences found for the ACC Synthase gene, *Ciclev10019920*, at 30 DPA, with similar expression levels, and at 260 DPA, when they are clearly higher in ARR, are in accordance with the role of ethylene in the promotion of color change, that in the early cultivar occurs in advance to CLE and HER [57].

In summary, the results of the qRT-PCR are coincident with the data obtained from RNA-Seq and support the hypothesis of the possible role of *Ciclev10021357* in the early and late ripening phenotypes of ARR and HER.

The presence of a hemizygotic 2 Mb deletion on chromosome 3, can explain the lower level of expression of *Ciclev10021357* in ARR. The same effect can be observed in the 225 genes present in the deleted region on chromosome 3, which would support the role of this large deletion on gene expression. It is worth noting that Nero, another somatic CLE mutation obtained with fast neutron mutagenesis, showing a fruit precocity phenotype strongly resembling that from ARR, displays a similar 2 Mb deletion of chromosome 3 [55]. The fact that the 2 somatic mutations derived from CLE share both the early phenotype and the same deletion on chromosome 3, that includes *Ciclev10021357* gene, supports the involvement of this gene in the precocious ripening in both cultivars.

The effect of large deletions over gene expression and the resulting phenotypes associated was previously reported for Clemenules mutants obtained by fast neutron mutagenesis, 39B3 and 39E7, that showed a delay in natural color break in fruit peel and carried DNA deletions in hemizygous dosage: there is a large deletion of 700 kb in 39B3, and at least two deletions of approximately 100 and 500 kb in 39E7 [72]. In grapevine, the deletion of a large region of at least 260 kb containing the regulatory genes VvMYBA1 and VvMYBA2, was described as the most likely cause of the alteration in the phenotype seen in Malian and Shalistin cultivars, that are bud sports of Cabernet Sauvignon bearing pale-colored berries [73].

## Conclusions

The time-course RNA-Seq study of citrus fruit ripening in the early-ripening ARR, the mid-season CLE and the late-ripening HER mutants of clementine evidenced that there is a strong correlation between the advancement/delay of the ripening process and a massive drift in gene expression implicating more than 5000 genes. These genes, that were grouped in different clusters based on their expression patterns, are involved in main processes that take place during fruit ripening such as size increase, color change, sugar accumulation, citric acid accumulation and decline, etc. Detailed analysis of transcription factors showed that most of the regulatory proteins with relevant roles in ripening were expressed in citrus fruits, disregarding they were described in climacteric or non-climacteric fruits. We also identified a SEP-like MADS box protein, *Ciclev10021357*, that could be negatively related with ethylene biosynthesis and therefore might be involved in the regulation of earliness during the ripening process of citrus fruits. In ARR, the presence of a hemizygous deletion on chromosome 3 might cause a reduction of the expression of *Ciclev10021357*, a circumstance that could accelerate the ripening rate of this cultivar. Furthermore, the differences in the expression of the genes responsible of ethylene biosynthesis, support the idea that the classic distinction between climacteric and non-climacteric patterns of ripening should be reviewed. Results from the present work suggest the relevance of the MADS-BOX TF on regulation of earliness and, therefore, further research would be performed to decipher its role on such relevant agricultural trait.

## Methods

### Plant material

Plant material was collected from 10 years old trees belonging to the IVIA cultivars collection. The 3 clementine (*Citrus clementina* Hort. ex Tan.) cultivars, Arrufatina, Clementina and Hernandina scions, clonally propagated, were grafted on Citrange Carrizo rootstock (*Citrus sinensis* (L.) Osb. X *Poncirus trifoliata* (L.) Raf).

### Samples collection and RNA extraction

Five fruit samples were collected from 2 different trees or biological replicas of each cultivar, as they were the only trees available in the same orchard, and we found it crucial to keep the same environmental conditions for all the trees, in order to avoid any unwanted effect over ripening. Samples were collected at different ripening states: 126, 154, 189, 240 and 275 DPE, accounting a total of 24 samples (Table 1). Pulp and peel were separated and stored at -80 °C until RNA extraction. Total RNA was isolated from frozen tissues using acid phenol extraction and Lithium Chloride precipitation method as described in Ecker and Davis 1987 [74]. PolyA RNA was isolated with RNEASY™ kit from Qiagen, following provider’s protocol. Purified polyA RNA was diluted in 100 μl of free RNAase water and quantified using Nanodrop.

### Illumina TruSeq™ RNA sequencing library preparation

Equal amounts of total RNA from pulp and peel were used for library construction. Pair-end Libraries were prepared using the TruSeq™ RNA sample preparation kit (Illumina Inc.,) according to manufacturer’s protocol. Briefly, 0.5 μg of total RNA was used for poly-A based mRNA enrichment selection using oligo-dT magnetic beads followed by fragmentation by divalent cations at elevated temperature resulting into fragments of 80-250 nt, with the major peak at 130 nt. First strand cDNA synthesis by random hexamers and reverse transcriptase was followed by the second strand cDNA synthesis performed using RNAseH and DNA Pol I. Double stranded cDNA was end repaired, 3’adenylated and the 3′- “T” nucleotide at the Illumina adaptor was used for the adaptor ligation. The ligation product was amplified with 15 cycles of PCR.

### Sequencing, base calling and quality trimming

Each pair-end library was sequenced using TruSeq SBS Kit v3-HS, in paired end mode with the read length 2x76bp. A minimum of 50 million paired end reads for each sample were generated on HiSeq2000 (Illumina, Inc) following the manufacturer’s protocol. Images analysis, base calling and quality scoring of the run were processed using the manufacturer’s software Real Time Analysis (RTA 1.13.48) and followed by generation of FASTQ sequence files by CASSAVA. Low quality bases with a Phred score lower than 13 (base-calling error probability limit = 0.05) were removed with CLC Genomics Workbench 7.0.3.

### RNA-Seq and differential expression analyses

RNA-Seq analysis was carried out by mapping sequencing reads and counting and distributing the reads across genes and transcripts with CLC-Bio Genomics Workbench 7.0.3 tool [75], with default parameters. The transcriptome [37] and the genome sequence of *C. clementina* [76] were used as reference for the mapping. Differential expression studies were carried out with EdgeR package [77] with *p*-values and FDR correction (0.05 cutoff).

Significant differential expression changes over time were assessed applying the R package named maSigPro, especially designed for dealing with RNA-Seq time series data [38, 78]. The package uses generalized linear models to evaluate the statistical significance and includes several tools to visualize the results.

A Multiple Series Time Course analysis was carried out with maSigPro, only with genes displaying more than 2 CPM in at least 2 samples, TMM method was used to normalize raw reads. FDR of 0.05 and R-Squared of 0.8 values were used as cut off to filter the results. Fuzzy C-Means Clustering was performed to obtain the clusters of co-expressed genes.

### Functional annotation

Blast2Go [39] was used for functional annotation of the longest transcript from each gene. Sequences were also searched for conserved proteins domains with IPRscan 5.0 [79] using the Blast2Go suite.

### Evolutionary relationships of proteins

Protein sequences were aligned with ClustalW [80], the evolutionary distances were computed using the Poisson correction method [81] and the evolutionary history was inferred using the Neighbor-Joining method [82] with bootstrap test with 500 replicates [83]. All Evolutionary analyses were conducted in MEGA7 [84].

### qRT-PCR analysis

Available total RNA from the original extractions from peel and newly extracted from whole fruitless were used to undergo gene expression analysis at 126 and 30 DPA, respectively. qRT-PCRs were performed using LightCycler® FastStart DNA MasterPLUS SYBR Green I reaction mix and a LightCycler 2.0 Instrument (Roche, Basel, Switzerland) to determine the relative mRNA levels in each total RNA extraction sample. The fluorescence intensity data was obtained through LightCycler Software version 4.1 and used to calculate the relative expression level of each gene through the ΔΔCt method using CitUBC1 as a housekeeping gene [85]. Total RNA extraction from Clemenules was used as a control. Specificity of the amplification reactions was assessed by melting temperature profiling of the amplicons yielded by each primer pair. The sequences of the forward and reverse primers and the size of the resulting fragments are listed in Additional file 10.

## Additional files

Additional file 1: **Table S1.** Sequencing and Read mapping summary (PDF 55 kb)

Additional file 2: **Table S2.** Functional annotation of the genes in the time-course clusters. (XLSX 205 kb)

Additional file 3: **Table S3.** Most abundant functional annotations in clusters (PDF 47 kb)

Additional file 4: **Table S4.** Genes involved in chlorophyll metabolism differentially expressed during ripening (XLSX 13 kb)

Additional file 5: **Table S5.** Genes involved in biosynthesis of carotenoids differentially expressed during ripening (XLSX 13 kb)

Additional file 6: **Table S6.** Genes involved in the TCA cycle differentially expressed during ripening (XLSX 28 kb)

Additional file 7: **Table S7.** TFs present in the time-course clusters Table S4. (XLSX 33 kb)

Additional file 8 **Table S8.** TFs differentially expressed in fruits (XLSX 262 kb)

Additional file 9: **Figure S1.** Image from the IGV genome browser showing the results of the BAM file with the reads alignment of CLE samples. Gray boxes show reads aligned to consensus sequence from *C. clementina*, thin blue lines join reads split by introns, indicating that they cover the exon junction. Transcripts are shown in the bottom panel. It becomes evident that *Ciclev10033176* and *Ciclev10033829* are really part of the same transcript and were wrongly annotated as different genes. (TIF 196 kb)

Additional file 10: **Table S9.** Primers used in the qRT-PCR assay. (DOCX 14 kb)

## Abbreviations

ACC: 1-aminocyclopropane − 1-carboxilic
ARR: Arrufatina
CLE: Clemenules
CPM: Count per Million
DPA: Days Post Anthesis
ERF: Ethylene responsive factor
FDR: False Discovery Rate
HER: Hernandina
TMM: Trimmed mean of M-values
