# Supplementary material for: Transcriptomic analysis of Citrus clementina mandarin fruits maturation reveals a MADS-box transcription factor that might be involved in the regulation of earliness
Source: BMC Plant Biol. 2019 Jan 31;19:47. doi: 10.1186/s12870-019-1651-z (PMC6357379; doi:10.1186/s12870-019-1651-z)
Supplement: Supplementary file 1 — Table S1. Sequencing and Read mapping summary (PDF 55 kb) [file 12870_2019_1651_MOESM1_ESM.pdf]

Table S1 Sequencing and Read mapping summary

Name	Number of reads	Number of reads after trim	Avg.length	Reads mapped in pairs	Reads mapped in broken pairs	Reads not mapped	Mapped Exon	Mapped Intron	Mapped Total gene
CL126R1	58241406	58161307	75.5	46164876	10334154	1584124	21070725	2011713	23082438
CL126R2	78555048	78447719	75.5	61529852	14584969	2229997	28000584	2764342	30764926
AR126R1	74320902	74181635	75.5	59055032	12919381	2071223	27043510	2484006	29527516
AR126R2	73134240	72702366	75.3	56351454	13130303	2792427	25324873	2850854	28175727
CL154R1	59208608	59114323	75.5	45764526	11591764	1667432	20903877	1978386	22882263
HE154R2	85066796	84906381	75.4	66580062	15303676	2862364	30464187	2825844	33290031
CL189R1	75767612	75576164	75.4	58766692	14314741	2303395	27035623	2347723	29383346
AR189R1	91148788	90805793	75.3	70923398	16734791	2804727	32477638	2984061	35461699
CL240R1	74622820	74493459	75.3	58669698	13413326	2281130	27150376	2184473	29334849
CL189R2	72054212	71895124	75.2	56809540	12634029	2292581	26057216	2347554	28404770
CL240R2	64561760	64435062	75.3	50850466	11310768	2147204	23437230	1988003	25425233
AR189R2	76403892	76254239	75.2	59737420	13846129	2521131	26920050	2948660	29868710
HE189R2	89022166	88840089	75.3	70297306	15870372	2490462	31840211	3308442	35148653
HE240R2	83646736	83563350	75.6	66156790	14852522	2470682	30504513	2573882	33078395
HE240R1	82277024	82172912	75.5	64354688	15148566	2565588	29767518	2409826	32177344
HE275R2	114485872	114301087	75.4	87996796	22457268	3662384	40652871	3345527	43998398
HE154R1	54932004	54875730	75.4	43240096	9881703	1697937	19605245	2014803	21620048
CL154R2	107318840	107202114	75.4	84162362	19547691	3375887	38211801	3869380	42081181
AR154R1	64409284	64327715	75.4	49530560	12622802	2093094	22871793	1893487	24765280
HE126R1	79278968	79135123	75.5	61686282	14749232	2556136	28382684	2460457	30843141
HE126R2	77586140	77518015	75.6	59998972	14602071	2849119	27059246	2940240	29999486
HE275R1	84153942	84016958	75,6	65469212	15405279	3006201	30064815	2669791	32734606
HE189R1	74150356	74051001	75,5	57906618	13384216	2661672	25837109	3116200	28953309
AR154R2	72442480	72344258	75,5	55828016	13772736	2645998	25375686	2538322	27914008
TOTAL	1866789896	1863321924	75.5	1457830714	342412489	59632895	666059381	62855976	728915357
