# Supplementary material for: Transcriptomic analysis of Citrus clementina mandarin fruits maturation reveals a MADS-box transcription factor that might be involved in the regulation of earliness
Source: BMC Plant Biol. 2019 Jan 31;19:47. doi: 10.1186/s12870-019-1651-z (PMC6357379; doi:10.1186/s12870-019-1651-z)
Supplement: Supplementary file 3 — Table S3. Most abundant functional annotations in clusters (PDF 47 kb) [file 12870_2019_1651_MOESM3_ESM.pdf]

Supporting Table S3 Most abundant functional annotations in clusters

GO Code	GO Term	Early	Late	Early-Late	Total
GO:0055114	oxidation-reduction process	30	180	137	347
GO:0006355	regulation of transcription, DNA-templated	26	156	129	311
GO:0009069	serine family amino acid metabolic process	18	112	69	199
GO:0009651	response to salt stress	7	78	59	144
GO:0009409	response to cold	10	49	30	89
GO:0009737	response to abscisic acid	5	29	40	74
GO:0005982	starch metabolic process	5	44	37	86
GO:0005985	sucrose metabolic process	4	40	34	78
GO:0009611	response to wounding	5	43	23	71
GO:0016567	protein ubiquitination	5	37	21	63
GO:0006979	response to oxidative stress	4	43	18	65
GO:0055085	transmembrane transport	8	27	31	66
GO:0009793	embryo development ending in seed dormancy	9	25	25	59
GO:0009414	response to water deprivation	10	27	26	63
GO:0006098	pentose-phosphate shunt	7	33	18	58
GO:0045893	positive regulation of transcription, DNA-templated	8	28	22	58
GO:0010200	response to chitin	5	30	22	57
GO:0032259	methylation	5	27	24	56
GO:0007165	signal transduction	5	29	20	54
GO:0006096	glycolytic process	5	29	20	54
GO:0009408	response to heat	4	25	17	46
GO:0006952	defense response	4	22	20	46
GO:0006094	gluconeogenesis	4	28	18	50
GO:0019288	isopentenyl diphosphate biosynthetic process, methylerythritol 4-phosphate pathway	3	28	18	49
GO:0009733	response to auxin	3	16	23	42
GO:0009744	response to sucrose	0	17	10	27
GO:0019252	starch biosynthetic process	2	15	15	32
GO:0009867	jasmonic acid mediated signaling pathway	0	16	12	28
GO:0009734	auxin-activated signaling pathway	1	10	10	21
GO:0009813	flavonoid biosynthetic process	0	12	10	22
	Total	202	1255	958	2415
