# Supplementary figures and images for: Transcriptomic analysis of Citrus clementina mandarin fruits maturation reveals a MADS-box transcription factor that might be involved in the regulation of earliness
Source: BMC Plant Biol. 2019 Jan 31;19:47. doi: 10.1186/s12870-019-1651-z (PMC6357379; doi:10.1186/s12870-019-1651-z)

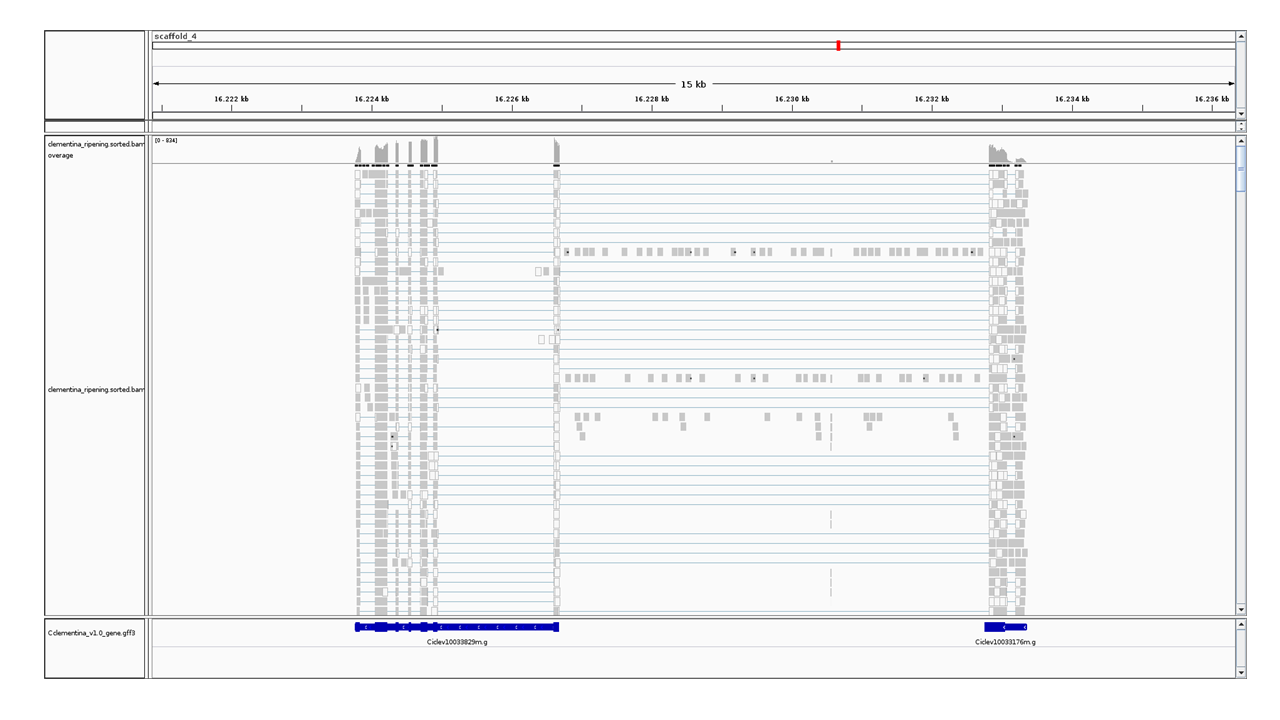

Supplement: Supplementary file 9 — Figure S1. Image from the IGV genome browser showing the results of the BAM file with the reads alignment of CLE samples. Gray boxes show reads aligned to consensus sequence from C. clementina, thin blue lines join reads split by introns, indicating that they cover the exon junction. Transcripts are shown in the bottom panel. It becomes evident that Ciclev10033176 and Ciclev10033829 are really part of the same transcript and were wrongly annotated as different genes. (TIF 196 kb) [file 12870_2019_1651_MOESM9_ESM.tif]
