# Supplementary material for: Transcriptomic analysis of Citrus clementina mandarin fruits maturation reveals a MADS-box transcription factor that might be involved in the regulation of earliness
Source: BMC Plant Biol. 2019 Jan 31;19:47. doi: 10.1186/s12870-019-1651-z (PMC6357379; doi:10.1186/s12870-019-1651-z)
Supplement: Supplementary file 10 — Table S9. Primers used in the qRT-PCR assay. (DOCX 14 kb) [file 12870_2019_1651_MOESM10_ESM.docx]

| **Table S9.** **Primers used in the qRT-PCR assay** | | | |
| --- | --- | --- | --- |
| Gene | Homolog | Primers | Product length (bp) |
| Ciclev10021357m | SlMADS1 | TTTCTCTAACCGTGGCAAGC/ | 97 |
|  |  | TTCGAGTGCACCAAAACTGC |  |
| Ciclev10032572m | RIN | TTGTGTGATGCTGAGGTTGC/ | 76 |
|  |  | TACTAGGGCTGCTGCAGAATTC |  |
| Ciclev10020575m | AGL65 | TTTCTTGGGCGCTGGTAAAG/ | 81 |
|  |  | TTCAAGTTGCCACCTCCATG |  |
| Ciclev10019920m | ACC synthase | AGCTGTGCTCGCAAAATGTC/ | 118 |
|  |  | TTTTAGCACTCTCGGCGATG |  |
| Ciclev10021100m | DOF | ACGCAAACGCAGACACAAAC/ | 149 |
|  |  | TGCACGGACACAGTTTCAAC |  |
